# OX40 agonism enhances PD-L1 checkpoint blockade by shifting the cytotoxic T cell differentiation spectrum

**DOI:** 10.1016/j.xcrm.2023.100939

**Published:** 2023-02-15

**Authors:** Tetje C. van der Sluis, Guillaume Beyrend, Esmé T.I. van der Gracht, Tamim Abdelaal, Simon P. Jochems, Robert A. Belderbos, Thomas H. Wesselink, Suzanne van Duikeren, Floortje J. van Haften, Anke Redeker, Laura F. Ouboter, Elham Beyranvand Nejad, Marcel Camps, Kees L.M.C. Franken, Margot M. Linssen, Peter Hohenstein, Noel F.C.C. de Miranda, Hailiang Mei, Adriaan D. Bins, John B.A.G. Haanen, Joachim G. Aerts, Ferry Ossendorp, Ramon Arens

**Affiliations:** 1 Department of Immunology, Leiden University Medical Center, 2333ZA Leiden, the Netherlands; 2 Department of Radiology, Leiden University Medical Center, 2333ZA Leiden, the Netherlands; 3 Systems and Biomedical Engineering Department, Faculty of Engineering, Cairo University, Giza 12613, Egypt; 4 Pattern Recognition and Bioinformatics, Delft University of Technology, 2628XE Delft, the Netherlands; 5 Department of Parasitology, Leiden University Center for Infectious Diseases, Leiden University Medical Center, 2333ZA Leiden, the Netherlands; 6 Department of Pulmonary Diseases, Erasmus Medical Center, 3015GD Rotterdam, the Netherlands; 7 Central Animal and Transgenic Facility, Leiden University Medical Center, 2333ZA Leiden, the Netherlands; 8 Department of Pathology, Leiden University Medical Center, 2333ZA Leiden, the Netherlands; 9 Department of Biomedical Data Sciences, Sequencing Analysis Support Core, Leiden University Medical Center, 2333ZA Leiden, the Netherlands; 10 Department of Internal Medicine, Amsterdam University Medical Center, 1105AZ Amsterdam, the Netherlands; 11 Division of Molecular Oncology and Immunology, Netherlands Cancer Institute, 1066CX Amsterdam, the Netherlands

**Keywords:** T cells, single-cell RNA sequencing, mass cytometry, systemic immune activation, immunotherapy, predictive biomarkers, immune checkpoint therapy

## Abstract

Immune checkpoint therapy (ICT) has the power to eradicate cancer, but the mechanisms that determine effective therapy-induced immune responses are not fully understood. Here, using high-dimensional single-cell profiling, we interrogate whether the landscape of T cell states in the peripheral blood predict responses to combinatorial targeting of the OX40 costimulatory and PD-1 inhibitory pathways. Single-cell RNA sequencing and mass cytometry expose systemic and dynamic activation states of therapy-responsive CD4^+^ and CD8^+^ T cells in tumor-bearing mice with expression of distinct natural killer (NK) cell receptors, granzymes, and chemokines/chemokine receptors. Moreover, similar NK cell receptor-expressing CD8^+^ T cells are also detected in the blood of immunotherapy-responsive cancer patients. Targeting the NK cell and chemokine receptors in tumor-bearing mice shows the functional importance of these receptors for therapy-induced anti-tumor immunity. These findings provide a better understanding of ICT and highlight the use and targeting of dynamic biomarkers on T cells to improve cancer immunotherapy.

## Highlights

• Sequential targeting of OX40 and PD-L1 results in superior tumor eradication
• OX40/PD-L1 targeting induces a systemic and dynamic effector T cell response
• OX40/PD-L1 facilitates circulating T cells co-expressing multiple NK cell receptors
• NK cell receptor expression on circulating T cells relates to tumoricidal activity

## Graphical abstract

**Figure:**
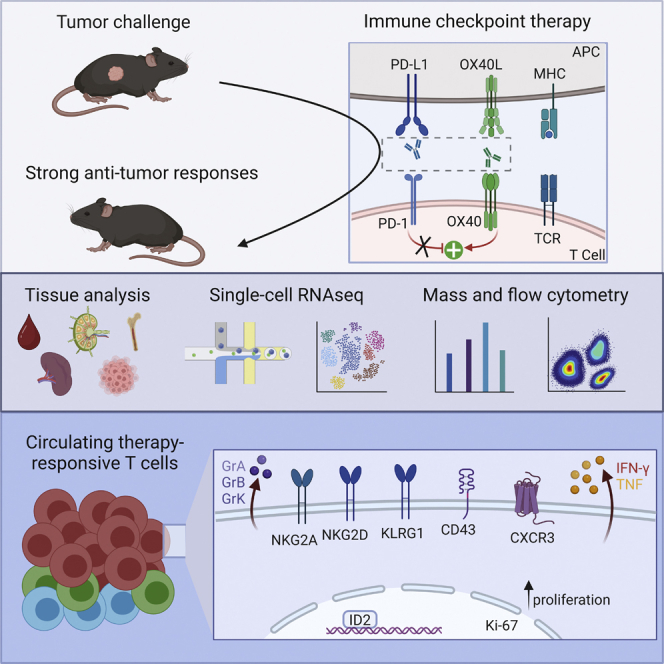

## Teaser

van der Sluis et al. demonstrate that immune checkpoint therapy by OX40/PD-L1 targeting induces circulating tumoricidal T cells that are highly proliferative, have cytotoxic potential, and co-express NK cell and chemokine receptors. The finding of these T cell subsets highlights their biomarker usage and targetability to improve clinical efficacy.

## Introduction

Immunotherapy has become an important treatment option for cancer patients but is only effective in a minority of patients. Therefore, a deeper understanding of factors governing immune responses upon immunotherapy is required to extend clinical efficacy to the majority of patients.^1^ Many studies have focused on characterizing intratumoral CD8^+^ T cells,^2^ but system-wide profiling studies have demonstrated that systemic anti-tumor immune responses are essential for immunotherapeutic efficacy.^3^ A comprehensive description of how effective cancer immunotherapy affects T cell states in the blood circulation is currently lacking.

Spontaneous regression of solid tumors is generally positively correlated with T cells infiltrating the tumor tissue.^4^ Expression of inhibitory molecules, including PD-1 and CTLA-4, on these T cells, however, is associated with impaired function, such as diminution of the cytotoxic and proliferative potential.^5^ Moreover, T cell costimulation is often diminished in tumor settings,^6^ leading to suboptimal T cell activation. To counteract cancer-associated T cell inhibition, successful immunotherapies called immune checkpoint therapy (ICT), were developed that block PD-1 and CTLA-4.^7^^,^^8^ Immune checkpoint blockade currently provides a recognized treatment option for several cancer types.^1^^,^^9^ However, response rates are still low, immune-related adverse events occur frequently, and long-term survival can only be achieved in a minority of patients,^10^ which warrants determination of the probability of a clinical response and development of more efficacious treatment options. In this respect, a better understanding of the cellular and molecular mechanisms that mediate tumor rejection could support the design of optimal treatment modalities.^11^ Moreover, predictive biomarkers related to effective therapy are highly desired, especially in light of numerous clinical trials with novel (combinatorial) immunotherapeutic approaches that are ongoing.^12^^,^^13^^,^^14^ In addition, methods directly targeting costimulatory receptors, such as members of the tumor necrosis factor receptor (TNFR) superfamily (e.g., CD27, CD134 [OX40], and CD137 [4-1BB]) expressed on tumor-specific T cells, have been developed and shown potential by itself and combined with immune checkpoint blockade.^15^^,^^16^ However, knowledge to support rational application of combinatorial ICT is lacking.

Emerging single-cell technologies, such as single-cell RNA sequencing (scRNA-seq) and high-dimensional flow and mass cytometry, have provided unprecedented insight into the heterogeneity of the tumor micro-environment (TME) and its modulation by immunotherapy.^17^^,^^18^^,^^19^^,^^20^^,^^21^ For example, these single-cell technologies highlight the identification of intratumoral T cells with different states of functionalities, ranging from cytotoxic to dysfunctional,^22^ and the existence of biomarkers in CD8^+^ T cells that are associated with responsive tumor regression.^23^ Predictive biomarkers in patients treated with ICT, such PD-1 and CTLA-4 blockade, have also been investigated in the systemic circulation, showing key roles of T cells,^24^^,^^25^^,^^26^^,^^27^^,^^28^^,^^29^^,^^30^^,^^31^^,^^32^^,^^33^ natural killer (NK) cells,^34^ and monocytes.^35^^,^^36^

Here, we performed deep profiling of the systemic T cell response induced by immunotherapeutic regimens built on driving agonist signals via OX40-mediated costimulation in conjunction with blocking of the inhibitory PD-1-PD-L1 pathway. The additive effects of combination therapy over monotherapy were assessed by studying the transcriptional and proteomic changes of therapy-responsive T cell populations in tumor-bearing mice using two complementary high-dimensional single-cell profiling techniques: scRNA-seq^20^ and mass cytometry.^37^ We found that combined ICT elicited the most profound impact on effector T cell states in the blood, characterized by dynamic kinetics and upregulation of specific biomarkers, including NK cell markers, cytotoxic molecules, and chemokine receptors. System-wide analysis revealed that therapy-responsive T cells were not limited to the blood but connected to other key immune compartments like the spleen, bone marrow, and tumor-draining lymph nodes. Analysis of human peripheral blood mononuclear cells obtained shortly after PD-1 therapy revealed similar effector T cell states in the blood that correlated with the clinical response rate. The identified biomarkers were functionally associated and implicated in treatment efficacy. This study reveals dynamic cellular changes occurring during effective ICT and the role of a set of biomarkers connected to the cytotoxic potential of CD8^+^ T cells, which are instrumental in tumor immunity and could be used to assess the level of immunotherapy efficiency.

## Results

### Identification of circulating immunotherapy-responsive T cell subsets by single-cell transcriptional profiling

**Figure 1:**
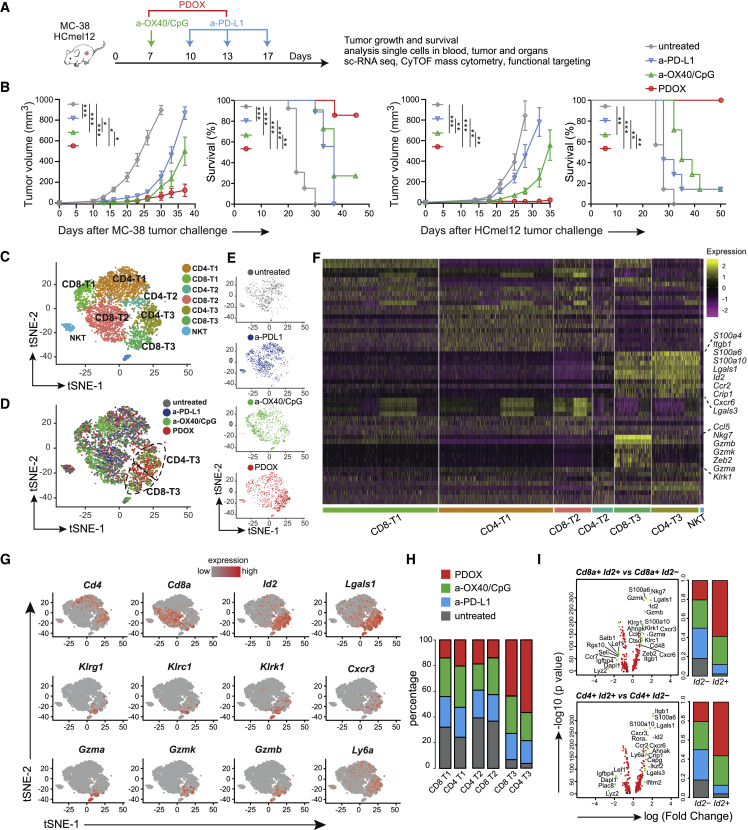
Transcriptional profiling identifies therapy-responsive T cell subsets in the blood circulation (A) Schematic of the immune checkpoint therapy (ICT) regimen strategy. Mice were challenged s.c. with MC-38 or HCmel12 syngenic tumors and treated with different ICTs. (B) MC-38 and HCmel12 tumor growth (mean ± SEM) and survival curves of untreated and anti-OX40/CpG-, anti-PD-L1-, and anti-OX40/CpG plus anti-PD-L1 (PDOX)-treated wild-type mice. Data were combined from two replicate experiments (n = 8–16 mice per group). ∗p < 0.05, ∗∗p < 0.01, ∗∗∗p < 0.001. (C) tSNE scRNA-seq plot visualizing six transcriptional clusters of blood T cells from day 18 tumor-bearing mice that were untreated or received ICT. (D) Combined t-SNE scRNA-seq plot of blood T cells color coded for the untreated and ICT groups. (E) tSNE scRNA-seq plots of blood T cells color coded for the untreated group and each ICT group individually. (F) Heatmap displaying scaled expression values of discriminative genes per cluster. (G) tSNE scRNA-seq plots displaying gene expression of *Cd4*, *Cd8*, *Id2*, *Lgals1*, *Klrg1*, *Klrc1*, *Klrk1*, *Cxcr3*, *Gzma*, *Gzmk*, *Gzmb*, and *Ly6a*. (H) Stacked bar graphs representing the percentage of cells from the untreated and ICT groups present in the six transcriptional clusters. (I) Volcano plots showing significant gene expression related to *Id2* expression in CD4^+^ and CD8^+^ T cells. Stacked bar graphs indicate the percentage of the cell origin according to their treatment. See also Figure S1.

To examine whether stimulating costimulatory receptors can improve PD-(L)1 checkpoint blockade, we challenged wild-type mice with syngeneic MC-38 tumors, which represents an ICT-sensitive colorectal cancer model. Tumor-bearing mice were subsequently treated with anti-PD-L1 antibodies, blocking the inhibitory PD-1/PD-L1 pathway, and with agonistic antibodies targeting the costimulatory receptor OX40 (Figure 1A). Blockade of the PD-1/PD-L1 axis resulted in delayed tumor outgrowth (Figure 1B), whereas anti-OX40 treatment did not delay tumor outgrowth (Figures S1A and S1B). Of note, addition of the TLR9 ligand CpG augmented the anti-tumoral activity of anti-OX40 (Figures S1A and S1B), which is in line with a previous report.^38^ On the other hand, CpG supplementation did not improve PD-L1 blockade (Figures S1C and S1D). Strikingly, the combination of PD-L1 blockade and anti-OX40/CpG treatment, referred to hereafter as PDOX, cured the majority of mice bearing MC-38 tumors (Figure 1B). This combination of immunotherapeutics was also most effective against established syngeneic HCmel12 melanoma tumors (Figure 1B).

On day 18 post MC-38 tumor challenge, the percentage of CD8^+^ and CD4^+^ T cells within the circulating leukocytes in blood and spleen increased after PDOX treatment compared with no treatment and anti-PD-L1 treatment (Figure S1E). The percentage of NK cells in the blood, however, remained similar but decreased in the spleen after PDOX treatment, while NK T cell percentages remained similar in the blood and spleen after any ICT. To identify the circulating T cell subsets associated with effective checkpoint therapy, we isolated CD4^+^ and CD8^+^ T cells from the peripheral blood on day 18 post MC-38 tumor challenge of treated (anti-PD-L1, anti-OX40/CpG, and PDOX) and untreated mice. Per condition, more than 1,000 cells were analyzed by scRNA-seq with a coverage of 60,000 reads per cell. The subpopulation structure of the circulating T cells was defined by pooling data from the different treatment groups, representing 5,600 cells in total, and using Seurat package analysis to identify transcriptional clusters. Six distinct T cell clusters could be identified, consisting of three CD4^+^ and three CD8^+^ T cell clusters (Figures 1C–1F). Two clusters (CD4-T3 and CD8-T3) were over-represented in the PDOX group (Figures 1D–1H), and both of these T3 clusters were characterized by expression of *Id2* and *Lgals1* transcripts encoding for the transcription factor ID2 and Galectin-1, respectively (Figures 1F and 1G). Other gene transcripts over-represented in the CD4-T3 and CD8-T3 clusters were *Cxcr3* (coding for the chemokine receptor CXCR3) and *Ly6a* (coding for Sca-1) (Figure 1G). Transcripts linked to NK cell receptors and cytotoxicity, including *Klrk1* (coding for NKG2D protein), *Klrc1* (coding for NKG2A), *Klrg1* (coding for KLRG1), and *Gzma*, *Gzmb*, and *Gzmk* (coding for Granzyme A, B, and K, respectively) were mostly enriched in CD8-T3 (Figure 1G).

Expression of ID2 as well as killer cell lectin receptor (KLR) family members are linked to cytotoxic effector CD8^+^ T cell differentiation, but their interconnectivity is unknown.^39^^,^^40^^,^^41^ To gain more insight into this connection of the *Id2*^*+*^, *Klkr1*^*+*^, and *Klrc1*^*+*^ subsets within the T3 clusters, the transcriptional profiles of these subsets within the CD8^+^ and CD4^+^ T cell lineages were analyzed independently (Figures 1I and S1F). Upregulation of *Id2* transcripts in CD8^+^ T cells was associated with increased expression of *Klrk1*, *Klrc1*, *Klrg1*, *Nkg7*, *Gzma*, *Gzmb*, *Gzmk*, *Zeb2*, *Lgals1*, *Cxcr3*, *Cxcr6*, *Cd48*, *Ctsd*, *Itgb1*, and *Ahnak*. The CD4^+^ *Id2*^+^ cells correspondingly upregulated *Lgals1*, *Cxcr3*, *Cxcr6*, *Itgb1*, and *Ahnak* and in addition upregulated *Lgals3*, *Ly6a*, *Ccr2*, *Capg*, *Crip1*, *Ifitm2*, *Ikzf2*, and *Rora*. Transcripts of *Dapl1*, *Lef1*, *Lyz2*, *Igfbp4*, and *Plac8* were consistently downregulated in CD8^+^ and CD4^+^ *Id2*^+^ T cells (Figure 1I). In the CD8^+^ and CD4^+^ T cell lineages, *Klrk1*^*+*^ and *Klrc1*^+^ cells showed highly similar expression (Figure S1F). Moreover, up- and downregulated transcripts were also shared with *Id2*^+^ cells, including *Lgals1*, *Gzma*, *Gzmb*, *Zeb2*, *Cxcr6*, *Ahnak*, *Ccr2*, *Nkg7*, *Dapl1*, and *Igfbp4*, which underscores the strong relationship between the KLR family, granzymes, and chemokines (Figure S1F). Altogether, these data indicate that combination therapy targeting OX40 and PD-L1 promotes expression of molecules associated with cytotoxicity and migration in responding CD8^+^ and CD4^+^ T cell subsets residing in the blood circulation.

### Circulating therapy-responsive T cell subsets display effector cell properties with increased cytotoxic and migratory capacity

**Figure 2:**
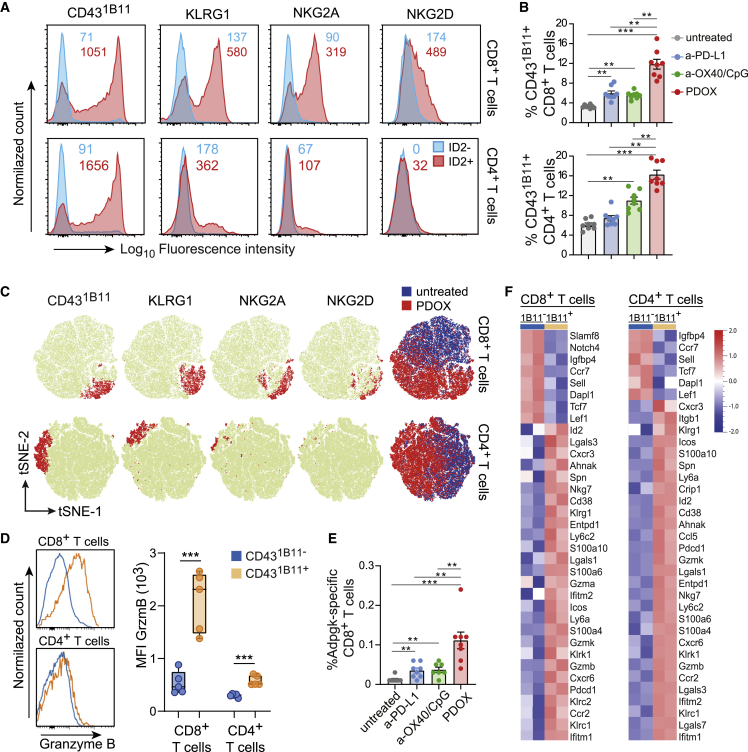
Circulating therapy-responsive T cell subsets display effector cell properties with increased cytotoxic and migratory capacity (A) Representative histogram plots of NKG2A, NKG2D, KLRG1, and CD43^1B11^ expression on gated ID2^−^CD8^+^ or ID2^+^CD8^+^ and gated ID2^−^CD4^+^ or ID2^+^CD4^+^ T cell populations residing in the blood circulation of MC-38-challenged PDOX-treated mice. Numbers indicate average mean fluorescence intensity. (B) Percentage of CD43^1B11+^ cells within the total CD8^+^ and CD4^+^ T cell population in the blood of untreated and ICT-treated groups. (C) tSNE plots of flow cytometric data visualizing NKG2A, NKG2D, KLRG1, and CD43^1B11^ expression (red) on CD8^+^ and CD4^+^ T cells in the blood from untreated and PDOX-treated groups. The blue/red tSNE plot indicates cell origin for CD8^+^ and CD4^+^ T cells of the untreated and PDOX-treated group, respectively. (D) Representative histograms (left) and quantification of fluorescence intensity (right) of granzyme B expression in blood circulating CD43^1B11−^CD8^+^ and CD43^1B11+^CD8^+^ and CD43^1B11−^CD4^+^ and CD43^1B11+^CD4^+^ T cell populations of MC-38-challenged PDOX-treated mice. (E) Percentage of Adpgk-specific CD8^+^ T cells in the blood of untreated and ICT-treated groups. (F) Heatmaps of RNA-seq data of sorted CD43^1B11−^CD8^+^ and CD43^1B11+^CD8^+^ and CD43^1B11−^CD4^+^and CD43^1B11+^CD4^+^ T cells (n = 2 individual mice per subset) from spleens isolated from wild-type mice challenged with MC-38 and treated with PDOX. Scaled expression values of discriminating genes are displayed. Data (A)–(F) are were collected from mice on day 18 post tumor challenge. The p values in (B) and (E) were calculated by ANOVA and in (D) by unpaired Student’s t test; ∗p < 0.05, ∗∗p < 0.01, ∗∗∗p < 0.001. Data in (B) and (E) are presented as mean ± SEM, and each dot in (B), (D), and (E) represents an individual mouse. Data (A)–(E) are representative of 2–3 independent experiments. See also Figure S2.

To validate the association of the *Id2* transcripts with transcripts of the *Klr* genes in subsets of the *Id2*^*+*^ cells at the protein level, the KLR family members and other effector T cell markers were co-stained with ID2 in circulating T cells obtained from tumor-challenged PDOX-treated mice. Within the ID2^+^CD8^+^ T cells, expression of KLRG1, NKG2A, and NKG2D was substantial, whereas ID2^−^CD8^+^ T cells lacked expression of these KLR family members. ID2^+^CD4^+^ T cells also expressed KLRG1 and NKG2A, albeit to a lesser extent as ID2^+^CD8^+^ T cells. ID2^−^CD4^+^ T cells were devoid of KLRG1 or NKG2A, while NKG2D was absent on ID2^+^CD4^+^ and ID2^−^CD4^+^ T cells (Figures 2A and S2A).

To further characterize the total ID2^+^ subset in CD4^+^ and CD8^+^ T cells, we examined other surface markers associated with NK cell receptors on effector T cells, including the O-glycan form of CD43 (sialoforine), which is known as a CD43 isoform expressed transiently by effector T cells.^42^ This activation-associated isoform of CD43 is generated by posttranslational glycosylation; hence, this form cannot be distinctively identified in scRNA analysis. However, the glycosylated form of CD43 can be visualized by the 1B11 antibody clone, while the antibody clone S11 recognizes CD43 regardless of glycosylation and reacts with virtually all T cells, activated or not (Figure S2B). Strikingly, the majority of ID2^+^CD8^+^ T cells expressed the hyperglycosylated CD43^1B11^ isoform, in contrast to ID2^−^CD8^+^ T cells, and similar results were found for ID2^+^CD4^+^ and ID2^−^CD4^+^ T cells (Figure 2A).

As expected from the connection with ID2 expression, CD43^1B11^-expressing CD8^+^ and CD4^+^ T cells were significantly increased upon PDOX treatment in the blood of MC-38- and HCmel12-challenged mice (Figures 2B and S2C). Cell-surface expression of NKG2A, NKG2D, and KLRG1 on peripheral blood T cells showed substantial overlap with CD43^1B11^ expression (Figure 2C). Accordingly, expression of NKG2A, NKG2D, and KLRG1 was increased on CD43^1B11+^ CD8^+^ T cells, and KLRG1 and NKG2A expression was increased on CD43^1B11+^ CD4^+^ T cells compared with their CD43^1B11−^ counterparts (Figure S2D). In line with the gene expression levels, CXCR3 and Ly6A (Sca-1) expression was increased on CD43^1B11+^ CD8^+^ and CD4^+^ T cells (Figure S2D). Furthermore, blood-circulating CD43^1B11^-expressing CD8^+^ but not CD4^+^ T cells from PDOX-treated mice expressed high levels of granzyme B (Figure 2D). Strikingly, tumor-specific CD8^+^ T cells recognizing the neo-epitope ASMTNMELM from *Adpgk*, expressed by MC-38 tumors,^43^ and the tumor antigens Trp2 and M8, expressed by HCmel12 tumors, were increased in the blood circulation, and these tumor-specific T cells highly expressed CD43^1B11^ (Figures 2E, S2E, and S2F).

To define and cross-validate the transcriptional signature of the circulating CD43^1B11+^ T cell subsets, we performed bulk mRNA sequencing on fluorescence-activated cell-sorted CD43^1B11−^ and CD43^1B11+^ CD8^+^ and CD43^1B11−^ and CD43^1B11+^ CD4^+^ T cells from MC-38 tumor-challenged mice that were treated with PDOX. Definitely, as also detected by scRNA-seq, the circulating CD43^1B11+^CD8^+^ and CD43^1B11+^CD4^+^ T cells had upregulated and downregulated genes analogous to the genes identified in the CD4-T3 and/or CD8-T3 clusters (e.g., *Id2*, *Lgals1*, *Klrc1*, *Klrg1*, *Klrk1*, *Ly6a*, *Cxcr3*, *Cxcr6*, *Ccr2*, *Gzma*, *Gzmb*, and *Gzmk*) (Figures 2F and S2G). Thus, the circulating PDOX therapy-responsive T cell subsets display induction of a transcriptional program that regulates the cytotoxic and migratory capacity.

### Dynamic induction of therapy-responsive T cell subsets

**Figure 3:**
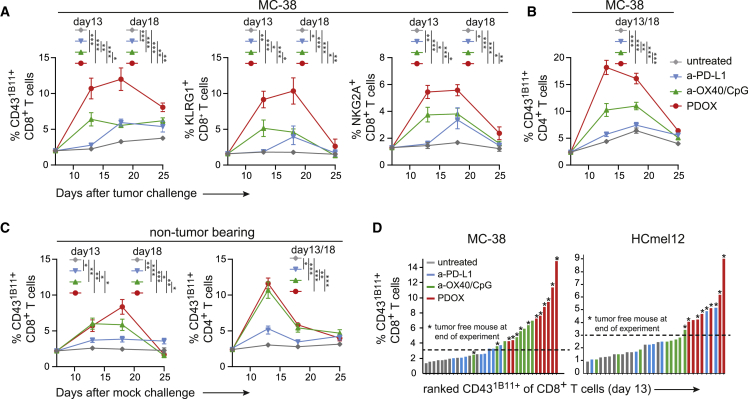
Dynamic induction of therapy-responsive T cell subsets in the blood circulation (A and B) Kinetics of the CD43^1B11+^, NKG2A^+^, and KLRG1^+^ cells of CD8^+^ T cells (A) and CD43^1B11+^ cells of CD4^+^ T cells (B) in the blood circulation after challenge with MC-38 tumor cells and treated or not treated with different ICTs (anti-PD-L1, anti-OX40, or PDOX). (C) Kinetics of the CD43^1B11+^ cells of CD8^+^ and CD4^+^ T cells after mock challenge (saline) and treated similarly as in (A) and (B). (D) Ranking of the percentage CD43^1B11+^ cells of CD8^+^ T cells in blood (on day 13 post tumor challenge) for each individual MC-38-bearing mouse (left panel) or HCmel12-bearing mouse (right panel). An asterisk indicates correlation with tumor-free mice. The p values in (A–C) were calculated by ANOVA; ∗p < 0.05, ∗∗p < 0.01, ∗∗∗p < 0.001. Data in (A)–(C) are presented as mean ± SEM Data shown in (A)–(D) are representative of 2–3 independent experiments.

To gain insight into the dynamics of the therapy-responsive T cell subsets, we longitudinally followed the CD43^1B11+^, NKG2A^+^, and KLRG1^+^ T cell subsets in the blood circulation of ICT-treated MC-38 tumor-bearing mice. Anti-OX40/CpG treatment, but not anti-PD-L1 treatment, increased the CD43^1B11+^CD8^+^ T cell subset on day 13 post tumor challenge (6 days after the start of the anti-OX40/CpG treatment), while at later time points these treatments resulted in a comparable increase compared with untreated mice. PDOX treatment elicited a much stronger increase in CD43^1B11+^CD8^+^ T cells on day 13 compared with anti-OX40/CpG and anti-PD-L1 treatment, and this increase was even more pronounced on day 18 (Figure 3A). On day 25 post tumor challenge, the percentage of CD43^1B11+^CD8^+^ T cells was decreased but still higher than in anti-OX40/CpG- and anti-PD-L1-treated mice. As projected based on the expression profile, the NKG2A^+^CD8^+^ and KLRG1^+^CD8^+^ T cell subsets showed similar kinetics (Figure 3A). PDOX treatment also induced the highest level of circulating CD43^1B11+^ CD4^+^ T cells on day 13 and day 18 post tumor challenge compared with other treatment groups (anti-OX40/CpG and anti-PD-L1) and untreated mice (Figure 3B).

To dissect the influence of the tumor on the dynamics of CD43^1B11+^CD8^+^ and CD43^1B11+^CD4^+^ T cells, we treated non-tumor-bearing mice (mock challenged) with anti-OX40/CpG, anti-PD-L1, or PDOX. While induction of CD43^1B11+^ T cells induced by the single therapies in the blood circulation were comparable between tumor-bearing and non-tumor-bearing mice, PDOX treatment amplified the response in tumor-bearing mice, indicating that tumor antigens and/or tumor-associated inflammation drives the synergy between anti-OX40/CpG and anti-PD-L1 blockade (Figure 3C).

A correlation between the level of CD43^1B11+^ CD8^+^ T cells circulating in the blood and MC-38 and HCmel12 tumor survival could be established (Figure 3D). Together, these data indicate that the therapy-elicited effector T cells, identified by the activation markers CD43^1B11^, NKG2A, and KLRG1, are characterized by dynamic expansion/contraction kinetics resembling those of vaccine- or infection-provoked T cell responses.

### System-wide induction of immunotherapy-responsive T cell subsets

**Figure 4:**
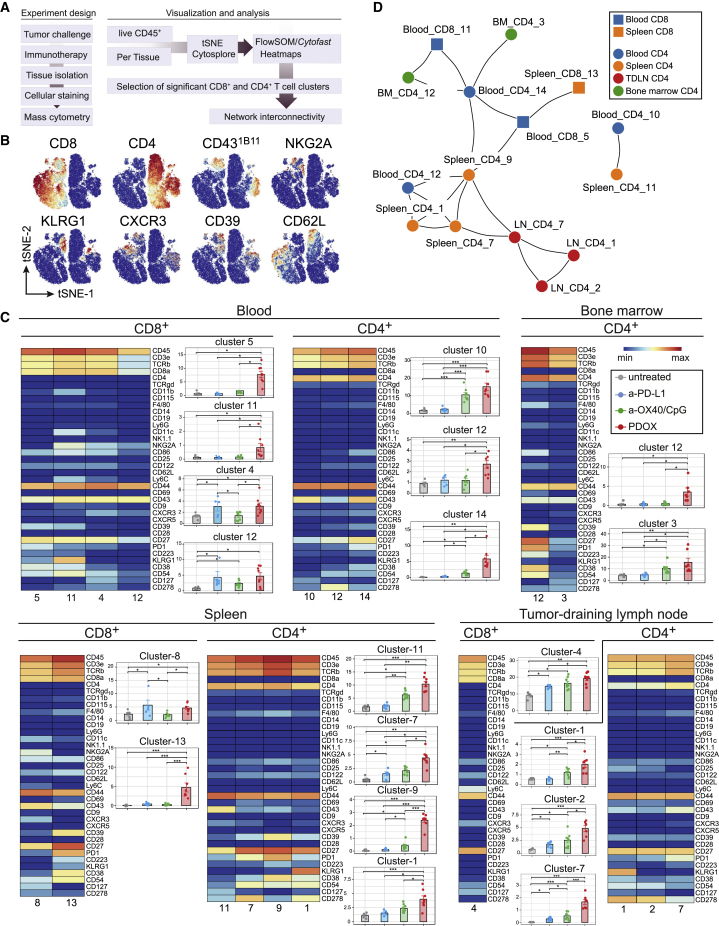
Systemic induction of therapy-responsive T cell subsets upon effective ICT (A) Schematic of the mass cytometry analysis of blood lymphocytes and lymphoid tissues. (B) tSNE plots of blood T cells isolated from tumor-bearing untreated and ICT-treated (anti-PD-L1, anti-OX40/CpG, PDOX) mice, visualizing the expression intensity of cell-surface markers measured by CyTOF mass cytometry. (C) Heatmaps of selected T cell clusters in the blood, bone marrow, spleen, and lymph nodes of untreated and ICT-treated mice. The level of ArcSinh5-transformed marker expression is displayed by a rainbow scale. Bar graphs indicate the abundance and significant differences of the selected T cell clusters. Data are represented as mean ± SEM, and each dot represents an individual mouse. The p values were calculated by ANOVA; ∗p < 0.05, ∗∗p < 0.01, ∗∗∗p < 0.001. (D) Network graph showing interconnectivity between CD4 (circle) and CD8 (square) T cell clusters in different compartments: blood (blue), spleen (orange), lymph nodes (red), and bone marrow (green). Highly correlated clusters (Pearson rho >0.8) are connected by lines, and cluster IDs are indicated. Data shown in (B)–(D) were collected from mice on day 18 post tumor challenge. See also Figure S3.

To interrogate whether the PDOX therapy-responsive effector T cell states were elicited system wide and whether these were interconnected, we assessed the phenotype of ICT-induced T cell states in lymphoid tissues on day 18 post MC-38 tumor challenge by CyTOF mass cytometry with 34 cell-surface markers, which allowed identification of T cell signatures in depth (Figure 4A). The marker panel included markers for effector T cell activation, differentiation, and migration, such as CD43^1B11^, NKG2A, KLRG1, CXCR3, and CD62L and the ectoenzymes CD38 and CD39 (Figure 4B). Viable CD4^+^ and CD8^+^ T cells were analyzed by hierarchical stochastic neighbor embedding (HSNE) using Cytosplore^44^ and Cytofast.^45^^,^^46^ We selected clusters based on the significant difference (p < 0.05) and abundance (>1%). Remarkably, in the blood compartment, all CD8^+^ and CD4^+^ T cell clusters that were significantly higher in PDOX-treated mice expressed CD43^1B11^ (Figures 4C and S3). Two of the four CD43^1B11+^CD8^+^ T cell clusters were most abundant in the PDOX-treated group compared with all other groups and co-expressed KLRG1, CD38, CD39, PD-1, or LAG-3 (blood cluster CD8-5) or co-expressed the same markers and NKG2A and ICOS (CD278) (blood cluster CD8-11). Three CD43^1B11+^CD4^+^ T cell clusters, which were more abundant in the PDOX-treated group compared with all other groups, co-expressed CXCR3 and ICOS and differentially expressed PD-1, CD38, and CD39. In the spleen, one CD8^+^ T cell cluster expressing CD43^1B11^ (spleen CD8-13) was most abundant in PDOX-treated mice, and, similar to blood cluster CD8-5, these cells co-expressed KLRG1, CD38, CD39, PD-1, and LAG-3. Four splenic CD4^+^ T cell clusters expressing CD43^1B11^ were higher in PDOX-treated mice and highly similar to the CD43^1B11^-expressing CD4^+^ T cells in the blood. In the tumor-draining lymph node (tdLN) and bone marrow, significantly higher frequencies of CD43^1B11+^CD4^+^ T cells but not CD43^1B11+^CD8^+^ T cells in the PDOX treated mice were detected, and these cells differentially expressed ICOS, CD38, CD39, and KLRG1 (Figures 4C and S3). Thus, besides also residing in the blood circulation in other lymphoid compartments, CD8^+^ and CD4^+^ T cell clusters expressing CD43^1B11^ and co-expressing NK cell receptors, chemokine receptors, and ectoenzymes are elicited that are connected to effective immunotherapy.

To determine the correlation between the identified therapy-responsive T cell clusters across all lymphoid tissues via an unbiased approach, correlation analyses were performed system wide (Figures 4D and S3). The therapy-responsive CD4^+^ and CD8^+^ T cell clusters in the blood are closely related to those in the spleen (*r* > 0.70), and blood CD4^+^ T cell clusters are also connected to the bone marrow CD4^+^ T cell clusters. Moreover, lymph node CD4^+^ T cell cluster 7 relates to splenic CD4^+^ T cell clusters. Together, these data show an interconnectivity between therapy-responsive T cell clusters residing in different lymphoid tissues, indicating induction of system-wide effects of ICT enabling efficient tumor immunity.

### Identification of NK cell receptors expressing CD8^+^ T cell subsets in the blood circulation of PD-1 therapy-responsive patients

**Figure 5:**
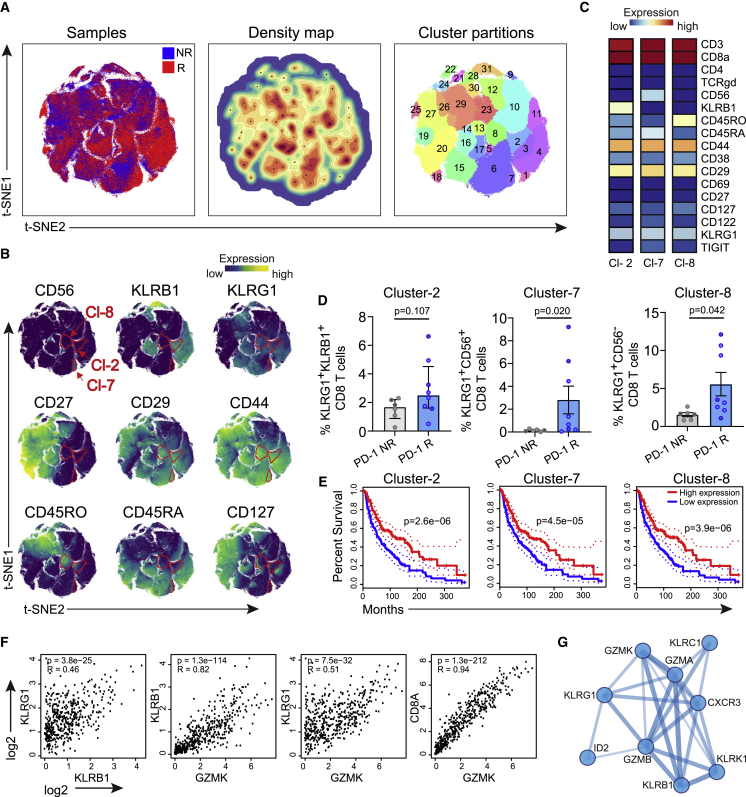
Identification of NK cell receptor-expressing CD8^+^ T cell subsets in the blood circulation of PD-1 therapy-responsive patients (A) Data-level tSNE plots of CD8^+^ T cells showing sample origin (left, including responding patients [red] and non-responding patients [blue]), a density map (center), and cluster partitions with numbering (right). See also Figures S4 and S5. (B) Expression intensity of specific cell surface markers. Color indication: blue, low expression; yellow, high expression. (C) Heatmaps of clusters 2, 7, and 8 displaying median marker expression values of a selection of markers. Color indication: blue, low expression; red, high expression. (D) Percentage of cells in clusters 2, 7, and 8 within the total CD8^+^ T cell pool. Data are from samples collected 2 week post PD-1 therapy and represented as mean ± SEM. Circles represent individual samples of non-responder melanoma (light gray), responder melanoma (light blue), non-responder lung cancer (dark gray), and responder lung cancer (dark blue) patients. The p values were calculated by Mann-Whitney U test. (E) Overall survival plots for high versus low gene signature expression of clusters 2, 7, and 8 for skin cutaneous melanoma (SKCM). Log rank p values are indicated. (F) Spearman correlation analysis of therapy-responsive marker genes for SKCM. Spearman correlation coefficient and p values are indicated. (G) Protein network analysis of the markers expressed by therapy-responsive T cells. Line thickness indicates strength of support for interaction.

To determine whether corresponding therapy-responsive T cell subsets could be identified in the blood circulation of patients receiving ICT, we evaluated anti-PD-1-responding and non-responding patients with melanoma or non-small cell lung cancer (NSCLC) by mass cytometry (Figures 5A and S4A–S4C). Peripheral blood was collected before and 2 weeks after treatment, and peripheral blood mononuclear cells (PBMCs) were stained with a panel of antibodies that incorporated detection of NK cell receptors expressed by activated human CD8^+^ T cells; i.e., KLRB1, KLRG1, and CD56 (Figure 5B). Data analysis by Cytosplore revealed distinct CD8^+^ T cell clusters that were increased in the anti-PD-1 responding group compared with the anti-PD-1 non-responders (Figures 5B–5D and S4C–S4F). These clusters expressed KLRG1, CD29, and CD44 and exclusively expressed KLRB1, CD56, or CD45RO. It is also worth noting that an additional CD8^+^ T cell cluster, also expressing the NK cell receptors KLRG1 and KLRB1, was elevated in 4 of 8 PD-1 responders (Figures S4D–S4F). To validate these observations, we performed FlowSOM clustering and projected these on opt-SNE dimensionality reduction plots (Figures S5A and S5B). Corroborating the Cytosplore-based data analysis, identical CD8^+^ T cell clusters were identified (i.e., positive for KLRG1, CD44, CD29, and CD56 or KLRB1) that were increased in the blood of PD-1-treated patients (Figures S5C and S5D).

Next, we evaluated the survival and correlation of the therapy-responsive gene signatures and marker genes discovered here in large cohorts of patients with skin cutaneous melanoma using Gene Expression Profiling Interactive Analysis.^47^^,^^48^ Higher expression of the gene signatures of clusters 2, 7, and 8 were related to better survival (Figure 5E). Higher expression of the KLR family members (*KLRB1*, *KLRG1*, *KLRC1*, and *KLRK1*), granzymes (*GZMB* and *GZMK*), as well as *CXCR3* and *ID2* also associated with a higher survival rate (Figure S5E). Correlation analysis of these genes indicated a strong association of the granzyme family with the KLR family members and with *CD8* (Figures 5F and S5F). Moreover, non-biased protein interaction analysis confirmed the connection between the therapy-responsive markers, indicating an underlying transcriptional program (Figure 5G). Thus, comparable with the findings in experimental settings, effective ICT in patients correlates with increases in CD8^+^ T cell subsets characterized by programming for cytotoxic effector function.

### Expansion of functional therapy-responsive CD8 T cell subsets in the TME and draining lymph nodes

**Figure 6:**
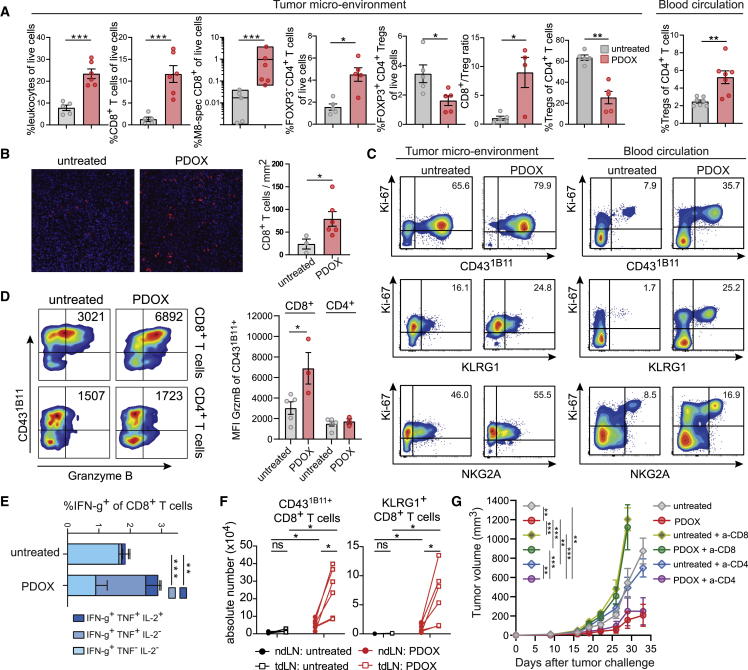
Expansion of the therapy-responsive CD8^+^ T cell subset in the blood circulation, TME, and draining lymph nodes (A) TME: percentage of leukocytes, CD8^+^ T cells, M8-specific CD8^+^ T cells, FOXP3^−^CD4^+^ T cells, and FOXP3^+^CD4^+^ Treg cell among live cells; CD8^+^ T cell/Treg cell ratio and percentage of Treg cells among total CD4^+^ T cells in the MC-38 tumor microenvironment (TME) of untreated and PDOX treated mice. Blood circulation: percentage of FoxP3^+^CD4^+^Treg cells among total CD4^+^ T cells in the blood of untreated and PDOX-treated mice. (B) Representative immunofluorescence images of MC-38 tumor-infiltrating CD8^+^ T cells (red) of untreated and PDOX-treated mice. The bar graph indicates absolute CD8^+^ T cell count per square millimeter. (C) Ki-67 expression versus CD43^1B11^, KLRG1, or NKG2A of CD8^+^ T cells in the TME and blood of untreated and PDOX-treated mice. Numbers indicate the average percentage of double-positive cells. (D) Left: representative flow cytometry plots indicating CD43^1B11^ versus granzyme B expression of MC-38 tumor-infiltrating CD8^+^ and CD4^+^ T cells. Numbers indicate the fluorescence intensity of granzyme B expression in CD43^1B11+^ T cells. Right: median fluorescence intensity of granzyme B expression in CD43^1B11+^CD8^+^ and CD43^1B11+^CD4^+^ T cells of untreated and PDOX-treated animals. (E) The proportion of single-, double-, and triple-cytokine-producing cells within the tumor-infiltrating CD8^+^ T cells of untreated and PDOX MC-38 tumor-challenged mice treated or not treated with PDOX. (F) Total numbers of CD43^1B11^ and KLRG1-positive CD8^+^ T cells in non-draining lymph nodes (ndLNs; closed circles) and tumor-draining lymph nodes (tdLNs; open squares). Lines connect ndLNs and tdLNs from the same mouse. (G) MC-38 tumor growth of untreated and PDOX-treated wild-type mice receiving CD8-depleting (yellow/green) and CD4-depleting (blue/purple) antibodies or mock. Data shown in (A)–(F) were collected from mice on day 20 post tumor challenge (PDOX treatment started on day 10). The p values in (A), (B), and (D)–(F) were calculated by unpaired Student’s t test and in (G) by ANOVA; ∗p < 0.05, ∗∗p < 0.01, ∗∗∗p < 0.001. Data in (A), (B), (D), (E), and (G) are presented as mean ± SEM, and each dot in (A), (B), and (D) represents an individual mouse. Data shown are representative of 2 independent experiments. See also Figure S6.

We next analyzed the therapy-responsive T cells in the TME of MC-38-challenged mice. Because PDOX treatment is very effective, treatment started at later time points (around day 10 with anti-OX40/CpG followed by PD-L1 blockade) to obtain sufficient tumor material for analysis. Compared with untreated animals, PDOX treatment increased the percentage of leukocytes in the TME, which was mainly caused by an increase in CD8^+^ T cells, and this correlated with an increase in tumor-specific CD8^+^ T cells (Figures 6A and S6A). In the TME of HCmel12-challenged mice, similar data were obtained (Figure S6B). Detection of CD8^+^ T cells by immunofluorescence showed that PDOX treatment promoted higher numbers of tumor-infiltrating CD8^+^ T cells (Figure 6B). The increase in CD8^+^ T cells coincided with a decrease in FOXP3^+^CD4^+^ regulatory T (Treg) cells; hence, the CD8^+^/Treg ratio was profoundly increased in the TME because of PDOX treatment (Figure 6A). The Treg cells in the peripheral blood were, however, relatively increased by the PDOX treatment. OX40 expression was not detected on intratumoral Treg cells upon PDOX therapy (Figure S6C), which can be attributed to obstruction of the injected anti-OX40 antibody and/or depletion of OX40^high^-expressing Treg cells.^49^^,^^50^ Like CD8^+^ T cells, helper CD4^+^ T cells (FOXP3^−^) were significantly increased in the TME (Figures 6A and S6A).

To assess the effector phenotype and potential of the tumor-infiltrated T cells, cell-surface expression, proliferation, granzyme expression, and cytokine production were evaluated. In untreated and PDOX-treated mice, the majority of tumor-infiltrated CD8^+^ T cells expressed CD43^1B11^, NKG2A, and/or KLRG1 (Figure S6D). The percentage of CD8^+^ T cells lacking any of these markers was decreased by PDOX treatment (p = 0.02, Mann-Whitney U test), indicating more activated CD8^+^ T cells in the TME. CD43^1B11^ and KLRG1 were also expressed by CD4^+^ T cells in the TME, and PDOX therapy mainly elevated CD43^1B11+^CD4^+^ T cells (Figure S6A). Moreover, the proliferation marker Ki-67 was abundantly expressed by tumor-infiltrating CD8^+^ T cells expressing CD43^1B11^, KLRG1, and NKG2A, whereas PDOX therapy only significantly increased KLRG1/Ki-67 double-positive cells (Figures 6C and S6E). Co-staining of Ki-67 with CD43^1B11^, KLRG1, and NKG2A of blood circulating CD8^+^ T cells, however, indicated a particular increase in Ki-67-co-expressing cells after PDOX therapy, suggesting that such marker combinations could be used as robust biomarkers (Figures 6C and S6E). A higher increase in Ki-67^+^CD43^1B11+^ and Ki-67^+^CD43^1B11+^ co-expression after therapy was also observed within circulating FoxP3^−^CD4^+^ T cell subsets compared with tumor-infiltrating FoxP3^−^CD4^+^ T cells (Figure S6E). Consistent with increased granzyme B levels in circulating CD8^+^ T cells after therapy (Figure 2D), granzyme B expression in CD43^1B11+^ tumor-infiltrating CD8^+^ T cells was increased by PDOX treatment (Figure 6D). PDOX therapy also elicited higher percentages of polyfunctional cytokine-producing CD8^+^ and CD4^+^ T cells (Figures 6E and S6F).

To better understand local and systemic immune responses, we compared tdLNs with non-draining lymph nodes (ndLNs). Here, we noticed that, in untreated animals, the tdLNs were enlarged compared with ndLNs (Figures 6F and S6G–S6I). PDOX therapy enhanced the absolute numbers in tdLNs and ndLNs and in particular increased the magnitude and percentages of CD43^1B11^- and KLRG1-expressing CD8^+^ and CD4^+^ T cells in the tdLNs (Figures 6F, S6G, and S6I–S6K). Accordingly, PDOX treatment also enhanced the magnitude of NKG2A- and NKG2D-expressing CD8^+^ T cells in tdLNs (Figures S6G, S6I, and S6J). In line with the increase in cytotoxic and cytokine polyfunctional CD8^+^ T cells, depletion of CD8^+^ T cells completely dismantled the efficacy of PDOX treatment (Figure 6G). Depletion of CD4^+^ T cells, despite being present in the TME and having an activated phenotype, did not impact tumor control in PDOX-treated mice (Figure 6G). Thus, PDOX-therapy-responsive CD8^+^ T cells are functionally effective and proliferate in the peripheral blood, TME, and draining lymph nodes.

### Functional receptor expression on therapy-responsive T cell subsets affects expansion and tumor infiltration

**Figure 7:**
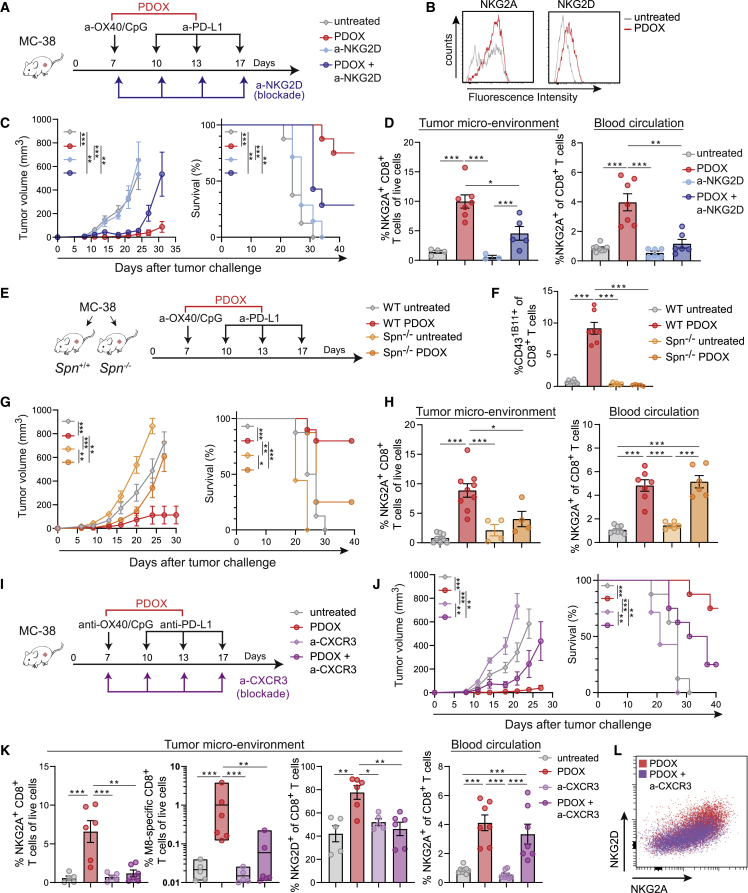
Functional expression of NKG2D, CD43, and CXCR3 on therapy-responsive T cell subsets affects expansion and tumor infiltration (A) Schematic of the strategy. Mice were challenged s.c. with MC-38 tumors and left untreated or treated with PDOX in combination with blocking NKG2D antibodies. (B) Representative histograms showing NKG2A and NKG2D expression on MC-38 tumor-infiltrating CD8^+^ T cells of PDOX-treated and untreated mice. (C) MC-38 tumor growth and survival curves of untreated and PDOX-treated mice in combination with blocking anti-NKG2D antibodies. (D) Percentage of NKG2A^+^ cells among blood CD8^+^ T cells and percentage of NKG2A^+^CD8^+^ T cells among live cells in the TME of untreated and PDOX-treated mice in combination with blocking NKG2D antibodies. (E) Schematic of the strategy. Wild-type and *Spn*^−/−^ mice were challenged s.c. with MC-38 tumors and left untreated or treated with PDOX. (F) Percentage CD43^1B11+^ cells among blood CD8^+^ T cells of untreated and PDOX-treated wild-type (WT) and *Spn*^−/−^ mice. (G) MC-38 tumor growth and survival curves of untreated and PDOX-treated WT and *Spn*^−/−^ mice. (H) Percentage of NKG2A^+^ cells among blood CD8^+^ T cells and percentage of NKG2A^+^CD8^+^ T cells among live cells in the TME of untreated and PDOX-treated WT and *Spn*^−/−^ mice. (I) Schematic of the strategy. WT mice were challenged s.c. with MC-38 tumors and left untreated or treated with PDOX in combination with blocking CXCR3 antibodies. (J) MC-38 tumor growth and survival curves of untreated and PDOX-treated mice in combination with CXCR3-blocking antibodies. (K) Left plots: percentage of NKG2A^+^ CD8^+^ T cells among live cells and percentage of M8-specific CD8^+^ T cells and percentage of NKG2D^+^ cells among CD8^+^ T cells in the TME of untreated and PDOX-treated mice in combination with blocking CXCR3 antibodies. Right plot: percentage of NKG2A^+^ cells among CD8^+^ T cells in the blood of untreated and PDOX-treated mice in combination with blocking CXCR3 antibodies. See also Figure S7. (L) Representative flow cytometry plot of NKG2A versus NKG2D expression of tumor-infiltrating CD8^+^ T cells in PDOX-treated mice in combination with blocking CXCR3 antibodies. Data shown in (B), (D), (F), (H), (K), and (L) were collected from mice on day 18 post tumor challenge. The p values in (C), (D), (F)–(H), (J), and (K) were calculated by ANOVA or log rank (survival); ∗p < 0.05, ∗∗p < 0.01, ∗∗∗p < 0.001. Data in (C), (D), (F)–(H), (J), and (K) are presented as mean ± SEM, and each dot in (D), (F), (H), and (K) represents an individual mouse. Data shown are representative of 2 independent experiments.

To functionally assess the relevance of the elevated levels of NK cell receptor expression on therapy-responsive CD8^+^ T cells, we targeted NKG2D, which is in contrast to the inhibitory NKG2A molecule known as a molecule with the capacity to provide costimulatory signals to T cells (Figure 7A).^51^ Like NKG2A, NKG2D is also upregulated in the TME (Figure 7B). Blockade of NKG2D reduced the effectiveness of the PDOX treatment in controlling tumor outgrowth, whereas NKG2D blockade in untreated mice had no implication (Figure 7C). This effect of NKG2D blockade on PDOX treatment was related to a diminution of NKG2A^+^CD8^+^ T cells in the blood circulation and TME (Figure 7D). Moreover, NK cell depletion did not impact tumor control of PDOX therapy, indicating that PDOX therapy relates primarily to CD8^+^ T cell-mediated effects (Figure S7A). We conclude that NK cell receptor-expressing T cell subsets are instrumental for PDOX therapeutic efficacy with NKG2D, supporting systemic stimulation of the therapy-responsive effector CD8^+^ T cells.

To functionally assess whether CD43 expression is critical for the efficacy of ICT, we examined PDOX responsiveness in settings of CD43 availability and absence. For this, wild-type mice and mice deficient in the *Spn* gene (coding for CD43) were challenged with MC-38 tumor cells and left untreated or treated with PDOX (Figures 7E and 7F). Whereas CD43 proficient mice showed the anticipated therapeutic efficacy of PDOX treatment upon tumor challenge, mice deficient in CD43 could not control MC-38 tumor outgrowth despite PDOX treatment (Figure 7G). Remarkably, CD43 deficiency did not affect the percentage of NKG2A^+^CD8^+^ T cells in the blood but rather resulted in a diminution of NKG2A^+^CD8^+^ T cells in the TME (Figure 7H), indicating that tumor migration of therapy-responsive T cells is regulated by CD43, which is in line with the ability of the CD43^1B11^ isoform to function as a ligand for the cell adhesion molecule E-selectin (CD62E).^52^ ID2 expression in tumor-infiltrating CD8^+^ T cells was not affected by PDOX treatment in wild-type or CD43-deficient mice (Figure S7B).

To test whether the chemokine receptor CXCR3, expressed on the CD43^1B11+^T cell subset and known to mediate adhesion induction,^53^ was also implicated in tumor migration, we blocked this chemokine receptor by antibodies provided during PDOX treatment of tumor-challenged mice (Figure 7I). Obstruction of CXCR3 resulted in reduced efficacy of PDOX to control tumor outgrowth (Figure 7J). This effect of CXCR3 blockade was related to decreased infiltration of CD43^1B11+^CD8^+^ T cells in the TME as well as reduced tumor-infiltrating NKG2A^+^CD8^+^ T cells and tumor-specific CD8^+^ T cells, while the CD43^1B11+^CD8^+^ and NKG2A^+^CD8^+^ T cells were unaffected in the blood circulation (Figures 7K and S7C). Moreover, CXCR3 blockade prevented NKG2D^+^CD8^+^ T cell entry into to the TME (Figures 7K and 7L). Altogether, we conclude that CD43 and CXCR3 mediate tumor migration of therapy-responsive CD8^+^ T cells, which is critical for these cells to achieve tumor control.

## Discussion

A major challenge for ICT is to overcome the substantial variability of this therapy through identification of predictive biomarkers. Ideally, such biomarkers can be interrogated in easily accessible compartments, such as the peripheral blood, while accurately reporting therapy responses in the TME. Here, we demonstrated, in two different murine tumor models, MC-38 (colorectal carcinoma) and HCmel12 (melanoma), that a dynamic and systemic T cell response develops upon efficient immunotherapy, which is characterized by an interconnected gene signature related to cytotoxicity and migration. Analyses of peripheral blood samples from PD-1 blockade therapy-responsive and unresponsive patients highlight the potential clinical utility of the cytotoxic gene signature consisting of several NK cell markers expressed by CD8^+^ T cells.

Combination of a PD-1/PD-L1 pathway antagonist and an OX40 agonist with CpG was remarkably efficient in eradicating developing tumors. These data show the possible benefit of using combinatorial treatment of already used therapeutics in patients (e.g., anti-OX40^54^ and anti-PD-L1^55^) and emphasize the potential of this combination, as has also been observed in other mouse tumor models.^56^^,^^57^ This harmonizing effect of the combinatorial treatment was deciphered by complementary high-dimensional single-cell technology platforms. scRNA-seq and mass cytometry highlighted functionally dynamic CD4^+^ and CD8^+^ T cell states that were characterized by NK cell receptor expression and expression of adhesion/migration receptors. The kinetics of the therapy-responsive T cells (i.e., sharp expansion followed by a contraction phase, which is typical for acute infection) may reflect temporal systemic activation, but T cell activation may be ongoing in the TME. Although less impressive, this expansion was also observed following OX40/CpG treatment, while PD-L1 blockade seems to mainly facilitate this T cell expansion in a combination setting. Additional PD-1 upregulation occurring following OX40 triggering may thus be efficiently counteracted and allows T cells to rapidly expand and differentiate to cytotoxic effector T cells. Although expression of OX40 on CD4^+^ T cells is higher compared with CD8^+^ T cells, this mechanism is likely to occur in CD4^+^ and CD8^+^ T cells because direct triggering of OX40 on CD4^+^ and CD8^+^ T cells results in effector T cell formation.^58^^,^^59^ OX40-activated CD4^+^ T cells may additionally help the CD8^+^ T cells with their expansion and differentiation.^60^ Nevertheless, we observed that depletion of CD4^+^ T cells had no effect on tumor control by PDOX treatment, which could be explained by depleting CD4^+^ helper T cells and inhibitory Treg cells. Another mechanism that could potentiate the PDOX combination may be related to intensification in OX40 expression on responding T cells after targeting of the PD-1/PD-L1 pathway.^61^^,^^62^^,^^63^ The addition of CpG to OX40 was synergistic, and this is likely related to enhanced upregulation of OX40 on T cells because of enhanced cytokine secretion by macrophages and dendritic cells.^38^ CpG is also able to upregulate costimulatory molecules such as CD70, CD80, and CD86,^64^ which may empower OX40-mediated costimulation.^65^ Although CpG did not enhance PD-L1 blockade in the subcutaneous (s.c.) administration setting we used, intratumorally provided CpG may synergize with blockade of the PD-1-PD-L1 pathway^66^ by enhancing dendritic-cell-mediated cross-presentation of tumor antigens.^67^ Whether triggering of OX40-related receptors, such as 4-1BB or CD27, belonging to the costimulatory members of the TNFR superfamily, has similar effects as described here remains to be determined.

Our study also indicated that the efficiency of the immunotherapeutic treatment is mirrored by the induction of peripheral T cells that could be identified by cell surface markers. The cell-surface expression of the hyperglycosylated form of CD43 and the NK cell receptors NKG2A, NKG2D, and KLRG1 on CD8^+^ T cells was a strong signature for these cells as an indicator for cytotoxic effector function based on the co-expression with granzymes. In line with this are findings in the TME of melanoma patients, where KLRG1 was found to be expressed in the cytotoxic T cell compartment.^22^ Moreover, fate-tracking studies in mice indicated that KLRG1^+^ CD8^+^ T cells display developmental plasticity and that basically these cells can differentiate into all memory T cell lineages, which underscores the value of this marker.^68^ Indeed, KLRG1^+^CD8^+^ T cells are excellent predictors of the effectivity of cancer vaccines.^69^ The combination of KLRG1 with the proliferation marker Ki-67 may provide an even better biomarker for circulating CD8^+^ T cells responding to immunotherapy. In the PBMC compartment of patients we also observed KLRB1 (CD161), which was recently discovered as a functional marker on human CD8^+^ T cells in the TME of glioma.^70^ Besides effects on CD8^+^ T cells, we also observed system-wide expansion and contraction of CD4^+^ T cells expressing CD43^1B11^, KLRG1, and CXCR3, which is in line with studies of human peripheral blood of anti-PD-1-treated patients in which CXCR3^+^CD4^+^ T cells correlated with a positive clinical outcome.^71^ Remarkably, transcripts of encoding NK cell receptors (*Klrc1*, *Klrk1*, and *Klrg1*) were observed in CD4^+^ T cells, but at the protein level only KLRG1 was found to be highly expressed, suggesting differential posttranscriptional regulation. Whether NKG2A and NKG2D, which likely have opposing functions with respect to T cell activation, are both concurrently functional is complex, given that the ligands of these receptors (i.e., HLA-E, and MICA/B, ULBP1-6, respectively) can be inducibly expressed in healthy and cancer tissue.^72^^,^^73^

The blocking studies targeting the NK cell receptor NKG2D and the chemokine receptor CXCR3, together with the CD43-deficient setting, identified the importance of simultaneous induction of cytotoxic and migratory properties. CD43 has also been involved in T cell activation, where its effects could be either costimulatory or negative regulatory;^74^^,^^75^^,^^76^ it may thus be determined spatiotemporally when and where CD43 exerts its pleiotropic effects. Although the combination therapy we used was already effective, blockade of NKG2A in more resistant tumors, which is known to synergize with PD-1 blockade,^77^ may further improve anti-tumoral responses. Another upregulated molecule upon PDOX treatment that could be targeted to improve outcome is the costimulatory molecule ICOS, which is linked to enhancing PD-1-targeted immunotherapy in mice^78^ but also in responsive patients.^79^

Additional clinical studies are required to determine the predictive significance of our findings. Studies with agonistic antibodies targeting costimulatory receptors such as OX40 in combination with CpG and inhibitory immune checkpoint blockade may be of particular interest. Recent studies already indicated the correlation between effector/effector-memory CD8^+^ T cell responses in the peripheral blood and clinical responses to immune checkpoint blockade.^29^^,^^32^^,^^33^ Our work highlights these studies and additionally proposes that effective combinatorial therapy is more powerful in induction of such peripheral T cell responses. Moreover, we show that NK cell receptors and the chemokine receptor CXCR3 are, besides biomarkers, also functional markers, and the targeting thereof may further improve the clinical response. Detection and targeting of glycosylated CD43 isoforms in ICT-treated patients may have therapeutic potential as well.

In conclusion, we provided evidence of an immune signature that relates to effective immunotherapy. Future studies entailing a systematic and multicenter cohort of patients with different cancer types for which a combinatorial anti-TNFR family member with anti-PD-1/PD-L1 treatment is approved remains needed. A prediction signature might then be directly used in clinical practice to stratify different levels of effectiveness of treatments.

### Limitations of the study

Although the PDOX treatment has been analyzed extensively here in the mouse models, this treatment was not tested in a clinical trial. In addition, the number of PD-1-treated patients included in the study is limited, and kinetics of human CD8^+^ T cell responses were not assessed. Furthermore, it remains unclear whether the impact of OX40 agonism can be solely attributed to enhancing the CD8^+^ T cells because depletion of CD4^+^ T cells did not abrogate PDOX efficacy. A direct impact on helper CD4^+^ T cells was observed but could have been counteracted by depletion of Treg cells in the circulation and in the tumor. The reduction in Treg cells in the tumor after PDOX therapy could be partly caused by the depletion effect of OX86, which may enhance the effectiveness of the PDOX therapy.

## STAR★Methods

### Key resources table

**Table:** 

REAGENT or RESOURCE	SOURCE	IDENTIFIER
**Antibodies**		

Anti-human/mouse Granzyme B PE/Cyanine7 (Clone QA16A02)	Biolegend	Cat# 372213; RRID: AB_2728381
Anti-mouse ID2eFluor 450 (clone ILCID2)	Thermo Fisher	Cat# 48-9475-82; RRID:AB_2735053
Anti-mouse NKG2D APC (clone CX5)	Thermo Fisher	Cat# 17-5882-81; RRID: AB_469463
Anti-mouse NKG2D PE-Cy7(clone CX5)	Thermo Fisher	Cat# 25-5882-82; RRID: AB_469657
Anti-mouse CD3 PerCP-Cy5.5 (clone 145-2C11)	Biolegend	Cat# 100327; RRID: AB_893320
Anti-mouse CD3 Pe-Fire700 (clone 17A2)	Biolegend	Cat# 100271; RRID: AB_2876394
Anti-mouse CD3 BV711 (Clone 17A2)	Biolegend	Cat# 100241; RRID: AB_2563945
Anti-mouse CD3 BV510 (clone 145-2C11)	BD Biosciences	Cat# 563024; RRID: AB_2737959
Anti-mouse CD3 BV785 (Clone 17A2)	Biolegend	Cat# 100231; RRID: AB_11218805
Anti-mouse CXCR3 BV650 (Clone CXCR3-173)	Biolegend	Cat# 126531; RRID: AB_2563160
Anti-mouse CXCR3 APC-Fire750 (Clone CXCR3-173)	Biolegend	Cat# 126539; RRID: AB_2650829
Anti-mouse CD43 PE (clone 1B11)	Biolegend	Cat# 121208; RRID: AB_493388
Anti-mouse CD43 PE-Dazzle594 (clone 1B11)	Biolegend	Cat# 121225; RRID: AB_2687245
Anti-mouse CD43 PE-Cy7 (clone 1B11)	Biolegend	Cat# 121218; RRID: AB_528813
Anti-mouse CD43 PE (clone S11)	Biolegend	Cat# 143205; RRID: AB_11142681
Anti-mouse CD8 BV421 (clone 53-6.7)	Biolegend	Cat# 100753; RRID: AB_2562558
Anti-mouse CD8 BUV395 (clone 53-6.7)	BD Biosciences	Cat# 563786; RRID: AB_2732919
Anti-mouse CD8 Super Bright 436 (clone 53-6.7)	Thermo Fisher	Cat# 62-0081-82; RRID: AB_2716981
Anti-mouse CD8 APC-R700 (clone 53-6.7)	BD Biosciences	Cat# 564983; RRID: AB_2739032
Anti-human/mouse CD44 BV785 (clone IM7)	Biolegend	Cat# 103059; RRID: AB_2571953
Anti-mouse Sca1 BV605 (clone D7)	Biolegend	Cat# 108134; RRID: AB_2650926
Anti-mouse CD25 BV480 (clone	BD Biosciences	Cat# 566120; RRID: AB_2739522
Anti-mouse KLRG1 BV605 (clone 2F1)	Biolegend	Cat# 138419; RRID: AB_2563357
Anti-mouse KLRG1 Pe-Cy7 (clone 2F1)	Thermo Fisher	Cat# 25-5893-82; RRID: AB_1518768
Anti-mouse KLRG1 BV785 (clone 2F1)	Biolegend	Cat# 138429; RRID: AB_2629749
Anti-mouse CD45 APC-fire 810 (clone 30-F11)	Biolegend	Cat# 103174; RRID: AB_2860600
Anti-mouse CD45 BB515 (clone 30-F11)	BD Biosciences	Cat# 564590; RRID: AB_2738857
Anti-mouse CD45.2 PerCP-Cy5.5 (clone 104)	BD Biosciences	Cat# 552950; RRID: AB_394528
Anti-mouse CD62L BV421 (clone MEL-14)	Biolegend	Cat# 104435; RRID: AB_10900082
Anti-mouse CD62L BV510 (clone MEL-14)	Biolegend	Cat# 104441; RRID: AB_2561537
Anti-mouse NKG2a APC (clone 16a11)	Biolegend	Cat# 142808; RRID: AB_11124538
Anti-mouse NKG2a PE-Cy7 (clone 16a11)	Biolegend	Cat# 142810; RRID: AB_2728161
Anti-mouse NKG2a/c/e FITC (clone 20D5)	BD Biosciences	Cat# 550520; RRID: AB_393723
Anti-mouse CD19 SparkBlue550 (clone 6D5)	Biolegend	Cat# 115565; RRID: AB_2819827
Anti-mouse CD4 A700 (clone RM4-5)	Biolegend	Cat# 100536; RRID: AB_493701
Anti-mouse CD4 BV711 (clone RM4-5)	Biolegend	Cat# 100550; RRID: AB_2562099
Anti-mouse OX40 BV421 (clone OX-86)	Biolegend	Cat# 119411; RRID: AB_10962569
Anti-mouse IFNgamma APC (clone XMG1.2)	Thermo Fisher	Cat# 17-7311-82; RRID: AB_469504
Anti-mouse TNF alpha FITC (clone MP6-XT22)	Biolegend	Cat# 506304; RRID: AB_315425
Anti-mouse IL-2 PE (clone JES6-5H4)	Thermo Fisher	Cat# 12-7021-82; RRID: AB_466150
Anti-mouse Ki-67 BV605 (Clone 16A8)	Biolegend	Cat# 652413; RRID: AB_2562664
Anti-mouse FoxP3 V450 (clone FJK-16s)	Thermo Fisher	Cat# 48-5773-82; RRID: AB_1518812
Anti-mouse CD3e (clone 145-2C11) (172Yb)	Thermo Fisher	Cat# 16-0031-85; RRID: AB_468848
Anti-mouse CD4 (clone RMA45) (145ND)	Fluidigm	Cat# 3145002B; RRID: AB_2687832
Anti-mouse CD8a (clone 53-6.7) (168Er)	Fluidigm	Cat# 3168003B; RRID: AB_2811241
Anti-mouse CD9 (clone eBioKMC8) (148ND)	Thermo Fisher	Cat# 14-0091-82; RRID: AB_823138
Anti-mouse CD11b (clone M1/70) (154Sm)	Fluidigm	3154006B
Anti-mouse CD11c (clone N418) (167Er)	Thermo Fisher	Cat# 14-0114-85; RRID: AB_467116
Anti-mouse CD14 (clone Sa14-2) (156Gd)	BioLegend	Cat# 123321; RRID: AB_2563720
Anti-mouse CD19 (clone 6D5) (166Er)	Fluidigm	Cat# 3166015; RRID: AB_2687846
Anti-mouse CD25 (clone 3C7) (150ND)	Fluidigm	Cat# 3150002; RRID: AB_2687835
Anti-mouse CD27 (clone LG.3A10) (158Gd)	Thermo Fisher	Cat# 14-0272-82; RRID: AB_1210515
Anti-mouse CD28 (clone 37.51) (151Eu)	Fluidigm	3151005B
Anti-mouse CD38 (clone 90) (163Dy)	Thermo Fisher	Cat# 14-0381-85; RRID: AB_467220
Anti-mouse CD39 (clone 24DMS1) (152Sm)	Thermo Fisher	Cat# 16-0391-83; RRID: AB_1210685
Anti-mouse CD43 (clone 1B11) (115In)	BioLegend	Cat# 121202; RRID: AB_493382
Anti-mouse CD44 (clone IM7) (142ND)	Thermo Fisher	Cat# 14-0441-85; RRID: AB_467247
Anti-mouse CD45 (clone 30-F11) (89Y)	Fluidigm	Cat# 3089005B; RRID: AB_2651152
Anti-mouse ICAM-1 (clone YN1/1.7.4) (164Dy)	BioLegend	Cat# 116102; RRID: AB_313693
Anti-mouse L-selectin (clone MEL-14) (169Tm)	BioLegend	Cat# 104443; RRID: AB_2562802
Anti-mouse CD69 (clone H1.2F3) (143ND)	Fluidigm	Cat# 3143004B; RRID: AB_2827881
Anti-mouse B7-2 (clone GL-1) (171Yb)	Thermo Fisher	Cat# MA1-10299; RRID: AB_11152324
Anti-mouse CD115/c-FMS/CSF1R (clone AFS98) (144ND)	Fluidigm	Cat# 3144012B; RRID: AB_2895116
Anti-mouse CD122 (clone TM-b1) (155Gd)	eBiosciences	Cat# 14-1222-85; RRID: AB_467448
Anti-mouse CD127 (clone A7R34) (175Lu)	Fluidigm	3175006B
Anti-mouse NK1.1 (clone PK136) (170Er)	Thermo Fisher	Cat# 14-5941-82; RRID: AB_467736
Anti-mouse CXCR3 (clone CXCR3-173) (149Sm)	Biolegend	Cat# 126506; RRID: AB_1027650
Anti-mouse CXCR5 (clone L138D7) (153Eu)	Biolegend	Cat# 145502; RRID: AB_2561955
Anti-mouse LAG-3 (clone eBioC9B7W) (161Dy)	Thermo Fisher	Cat# 14-2231-85; RRID: AB_493950
Anti-mouse PDL1 (clone 10F.9G2) (160Gd)	BioLegend	Cat# 124302; RRID: AB_961228
Anti-mouse ICOS (clone C.3984A) (176Yb)	BioLegend	Cat# 313502; RRID: AB_416326
Anti-mouse PD1 (clone 29F.1A12) (159Tb)	Fluidigm	Cat# 3159024; RRID: AB_2687839
Anti-mouse F4/80 (clone BM8) (174Yb)	Thermo Fisher	Cat# 14-4801-85; RRID: AB_467559
Anti-mouse KLRG1 (clone 2F1) (162Dy)	Thermo Fisher	Cat# 16-5893-85; RRID: AB_469132
Anti-mouse Ly6C (clone HK1.4) (165Ho)	Thermo Fisher	Cat# 16-5932-85; RRID: AB_2573096
Anti-mouse Ly6G (clone 1A8) (141Pr)	Fluidigm	Cat# 3141008B; RRID: AB_2814678
Anti-mouse MHCII (clone M5/114.15.2) (209Bi)	Thermo Fisher	Cat# 14-5321-85; RRID: AB_467562
Anti-mouse NKG2A (clone 20d5) (147Sm)	Thermo Fisher	Cat# 16-5896-85; RRID: AB_657831
Anti-mouse TCRab (clone H57-597) (173Yb)	ThermoFisher	Cat# 14-5961-85; RRID: AB_467759
Anti-mouse TCRgd (clone eBioGL3) (146ND)	Thermo Fisher	Cat# 14-5711-82; RRID: AB_467569
Anti-human CD38 (clone HIT2) (172Yb)	BioLegend	Cat# 303535; RRID:AB_2562819
Anti-human CD45RO (clone UCHL1) (164Dy)	Fluidigm	Cat# 3164007B; RRID: AB_2811092
Anti-human CD45RA (clone HI100) (166Er)	BioLegend	Cat# 304143; RRID: AB_2562822
Anti-mouse/human CD44 (clone IM7) (171Yb)	Fluidigm	Cat# 3171003B; RRID: AB_2895121
Anti-human CD45 (clone HI30) (89Y)	Fluidigm	Cat# 3089003; RRID: AB_2661851
Anti-human CD49b (clone P1E6-C5) (141Pr)	BioLegend	Cat# 359302; RRID: AB_2562682
Anti-human CD39 (clone A1) (161Dy)	BioLegend	Cat# 328221; RRID: AB_2563747
Anti-human HLA-DR (clone L243) (174Yb)	Fluidigm	Cat# 3174001; RRID: AB_2665397
Anti-human CD29 (clone TS2/16) (173Yb)	BioLegend	Cat# 303002; RRID: AB_314318
Anti-human CD11b (clone ICRF44) (209Bi)	Fluidigm	Cat# 3209003; RRID: AB_2687654
Anti-human TCRgd (clone 11F2) (115In)	Dianova/Thermo Fisher	Cat#; MUB1809P
Anti-human PD-L1 (clone 29E.2A3) (144ND)	Fluidigm	Cat# 3156026; RRID: AB_2687855
Anti-human CD8a (clone RPA-T8) (146ND)	Fluidigm	Cat# 3146001; RRID: AB_2687641
Anti-human ICOS (clone C398.4A) (151Eu)	Fluidigm	Cat#; 3151020B
Anti-human CD103 (clone Ber-ACT8) (152Sm)	BioLegend	Cat# 350202; RRID: AB_10639864
Anti-human CD49A (clone SR84) (155Gd)	BD Biosciences	Cat# 559594; RRID: AB_397287
Anti-human CD27 (clone L128) (167Er)	Fluidigm	Cat# 3167006B; RRID: AB_2811093
Anti-human CD127 (clone A019D5) (168Er)	Fluidigm	Cat# 3168017B; RRID: AB_2756425
Anti-human CD25 (clone 2A3) (169Tm)	Fluidigm	Cat# 3169003; RRID: AB_2661806
Anti-human CD3 (clone UCHT1) (170Er)	Fluidigm	Cat# 3170001B; RRID: AB_2811085
Anti-human CD4 (clone RPA-T4) (176Yb)	Fluidigm	Cat# 3176010B; RRID: AB_2810247
Anti-human TIGIT (clone MBSA43) (142ND)	Thermo Fisher	Cat# 16-9500-82; RRID: AB_10718831
Anti-human CXCR5 (clone MAB190) (143ND)	RnDsystems	Cat# MAB190; RRID: AB_2292654
Anti-human CD62L (clone DREG-56) (147Sm)	BioLegend	Cat# 304835; RRID: AB_2563758
Anti-human CD69 (clone FN50) (149Sm)	BioLegend	Cat# 310939; RRID: AB_2562827
Anti-human CD86 (clone IT2.2) (150ND)	Fluidigm	Cat# 3150020; RRID: AB_2687852
Anti-human CD154/CD40L (clone 24–31) (154Sm)	Thermo Fisher	Cat# 14-1548-82; RRID: AB_467520
Anti-human CD134/OX40 (clone ACT35) (158Gd)	BD Biosciences	Cat# 555836; RRID: AB_396159
Anti-human CD161 (clone HP-3G10) (159Tb)	Fluidigm	Cat# 3159004B; RRID: AB_2756421
Anti-human CD335/NKp46 (clone BAB281) (162Dy)	Fluidigm	Cat# 3162021B
Anti-human KLRG1 (clone SA231A2) (163Dy)	BioLegend	Cat# 367702; RRID: AB_2632728
Anti-human LAG-3 (clone 11C3C65) (165Ho)	Fluidigm	Cat# 3165037B; RRID: AB_2810971
Anti-human CD20 (clone 2H7) (106Cd)	BioLegend	Cat# 302343; RRID: AB_2562816
Anti-human CD14 (clone Tük4) (110Cd)	Thermo Fisher	Cat# MHCD1400; RRID:AB_10371749
Anti-human CD56 (clone 5.1H11) (111Cd)	BioLegend	Cat# 362502; RRID: AB_2563558
Anti-human CD150 (clone A12) (112Cd)	BioLegend	Cat# 306302; RRID: AB_314590
Anti-human CD244 (clone 2–69) (113Cd)	BioLegend	Cat# 393502; RRID: AB_2728427
Anti-human CD160 (clone BY55) (114Cd)	BioLegend	Cat# 341202; RRID: AB_2074411
Anti-human CCR6 (clone G034E3) (116Cd)	BioLegend	Cat# 353427; RRID: AB_2563725
Anti-human CD122 (clone Tu27) (145ND)	BioLegend	Cat# 339015; RRID: AB_2563712
Anti-human 4-1BB (clone 4B4-1) (148ND)	BioLegend	Cat# 309802; RRID: AB_314781
Anti-human CXCR6 (clone K041E5) (153Eu)	BioLegend	Cat# 356002; RRID: AB_2561738
Anti-human NKG2A (clone 131,411) (160Gd)	RnDsystems	Cat# MAB1059; RRID: AB_2280982
Anti-human PD-1 (clone EH12.2H7) (175Lu)	Fluidigm	Cat# 3175008; RRID: AB_2687629
MAb anti-mouse NK1.1 (clone PK136)	BioXcell	BE0036
MAb anti-mouse CD4 (clone GK1.5)	BioXcell	BE0003-1
MAb anti-mouse CXCR3 (CD183) (clone CXCR3-173)	BioXcell	BE0249
MAb anti-mouse NKG2D (CD314) (clone CX5)	BioXcell	BE0334
MAb anti-mouse CD8a (clone 2.43)	BioXcell	BE0061
Mab anti-mouse OX40 (clone OX86)	OX86 hybridoma culture	N/A
Mab anti-mouse PD-L1 (clone MIH5)	MIH5 hybridoma culture	N/A
rabbit anti-mouse CD8 (clone D4W2Z)	Cell signaling	98941T
Goat anti-rabbit AF647	Thermo Fisher	Cat# A32733; RRID: AB_2633282

**Chemicals, peptides, and recombinant proteins**		

7AAD	Thermo Fisher	Cat# A1310
Remel™ PHA Purified	ThermoFisher Scientific	R30852801

**Critical commercial assays**		

True-Nuclear™ Transcription Factor Buffer Set	Biolegend	424,401
FOXP3/Transcription factor STaining buffer set	eBioscience	00-5523-00
Nucleospin RNA Mini Kit	Macherey-Nagel	740,955.50
Zombie Aqua™ Fixable Viability Kit	Biolegend	Cat# 423102
LIVE/DEAD™ Fixable Blue dead cell stain kit	ThermoFisher	L23105
Pan T cell Isolation Kit I	N/A	N/A
Debris Removal Solution	Miltenyi Biotec	130-109-398

**Deposited data**		

CD3^+^ scRNAseq	https://www.ncbi.nlm.nih.gov/geo/	GEO: GSE193699

**Experimental models: Cell lines**		

Mouse: HCmel12 melanoma	Obtained from prof. T. Tüting	N/A
Mouse: MC38 colon carcinoma	Obtained from M.P. Colombo	N/A
OX86 hybridoma, anti-mouse OX40 mAb producing cells	Obtained from prof. M. Croft	N/A
MIH5 hybridoma, anti-mouse PD-L1 mAb producing cells	Obtained from M. Azuma	N/A

**Experimental models: Organisms/strains**		

Mouse: C57BL/6JRj	Janvier LABS	https://janvier-labs.com/en/fiche_produit/2_c57bl-6jrj_mouse/
Spn−/− mice (Spn^em1Lumc^)	Transgenic Facility Leiden, this manuscript.	MGI:6360988

**Oligonucleotides**		

CpG (ODN1826)	InvivoGen	Tlrl-1826

**Software and algorithms**		

FlowJo v10	Tree Star, Inc.	www.flowjo.com
Rv4.1.2 and R studio	R Consortium	https://www.rstudio.com/
OMIQ	Omiq Inc (CA, USA)	www.omiq.ai
Seurat version 2	R studio	https://satijalab.org/seurat/
Cytofast	Beyrend et al. 2018	https://rdrr.io/bioc/cytofast/
Cell ranger version 2.1.1	10x Genomics	https://support.10xgenomics.com/single-cell-gene-expression/software/downloads/latest
Qlucore Omics Explorer	Qlucore	https://qlucore.com/omics-explorer
GraphPad Prism V9.3.1	GraphPad Software	https://www.graphpad.com/
inForm V.2.4 image analysis software	PerkinElmer	N/A
Circos	Circos	http://circos.ca/
Igraph	Rstudio	https://igraph.org/
Cytosplore	Cytosplore	https://www.cytosplore.org/
MATLAB (R2016a (9.0.0.341360))	Mathworks	https://nl.mathworks.com/products/matlab.html
Gene Expression Profiling Interactive Analysis	GEPIA	http://gepia.cancer-pku.cn/
Gene Expression Profiling Interactive Analysis 2	GEPIA2	http://gepia2.cancer-pku.cn/#index

**Other**		

H-2Kb MuLV p15E Tetramer-KSPWFTTL (M8, gp70 604-611aa) - APC	LUMC Tetramer Facility	N/A
H-2Kb Trp2 Tetramer-SVYDFFVWL - APC	LUMC Tetramer Facility	N/A
H-2Db ADPGK Tetramer-ASMTNMELM - PE	LUMC Tetramer Facility	N/A

### Resource availability

#### Lead contact

Further information and requests for resources and reagents should be directed to the lead contact, Ramon Arens (r.arens@lumc.nl).

#### Materials availability

Mouse lines generated in this study are available upon request.

### Experimental model and subject details

#### Animal studies

C57BL/6 female and male mice were obtained from Janvier Laboratories (Le Genest-Saint-Isle, France). At the start of the experiments, mice were 6–8 weeks old. Mice were housed in groups of 2–5 animals in individually ventilated cages (IVC) under specific pathogen-free (SPF) conditions in the animal facility of Leiden University Medical Center (LUMC, Leiden, The Netherlands). All animal experiments were approved by the local and national committees of animal experiments under the permit numbers AVD116002015271, AVD116002015271 and AVD1160020186804, and performed according to the recommendations and guidelines set by the LUMC and by the Dutch Act on Animal Experimentation and EU Directive 2010/63/EU. Age and gender-matched mice were compared and randomly assigned to treatment groups.

#### Generation of *Spn (Cd43)* knockout mice

Spn^−/−^ mice (*Spn*^em1Lumc^; MGI:6360988) were generated using CRISPR-Cas9-mediated targeting of zygotes, which resulted in deletion of the coding sequence of Exon 2 of the *Spn* (*Cd43*) gene. C57BL/6J zygotes were microinjected at embryonic day 2 (E1.5, 2cell stage), with CRISPR/Cas9 RNP complexes with guide sequences CCTCAATCTCTATGAGCAAC and GGTGCAAGGCCATCTCCAGA and transferred to pseudo-pregnant recipients (crRNA, tracrRNA and Cas9 obtained from IDT). Mosaic candidates were selected based on PCR with primers upstream 5′ Crispr and downstream of the 3′ Crispr, followed by Sanger sequencing of the PCR product. One founder animal was selected with a deletion of Chr7: 126,735,256-126,736,568 (GRCm39) encompassing the complete coding sequence of the gene. The line was maintained on a C57BL/J6 background. PCR and sanger sequencing analysis of 5 most likely off-target sites based on CRISPOR.tefor.net scores, showed no Off-targets events (data not shown).

#### Cancer patient samples

Human peripheral blood mononuclear cells (PBMCs) from melanoma and non-small cell lung cancer (NSCLC) patients treated with PD-1 antibodies (pembrolizumab or nivolumab) were collected at the Amsterdam University Medical Center, the Netherlands Cancer Institute and Erasmus MC. PBMCs were isolated and cryopreserved in medium after Ficoll density gradient centrifugation. All samples were thawed on the same day, stained with the same antibody mixture and subsequently analyzed. Internal PBMC controls were routinely used to check the reproducibility of the acquisition. Ethical approval was provided by the local medical ethical committees, and written informed consent was obtained in accordance with the Declaration of Helsinki. Details on tumor type, age, gender and response to therapy are provided in Figure S4.

#### Cell lines and tumor challenge models

MC-38 cells were cultured in IMDM medium (Lonza) supplemented with Fetal Calf Serum (Greiner, 2mM L-glutamine (Gibco), 100 IU/mL Penicillin/streptomycin (Gibco) and 25 μM 2-mercaptoethanol. Experiments performed with the MC-38 cell line,^80^ established from a tumor arisen from female mice, were performed with female mice. HCmel12 cells were cultured in RPMI supplemented with 8% FCS, 2mM L-glutamine, 10 nm NEAA, Sodium pyruvate, 1mM HEPES and 20 μM 2mercaptoethanol, 100 IU/mL penicillin/streptomycin (Gibco). Experiments performed with HCmel12,^81^ which originated from a male mouse, were performed in male mice. Hybridoma cells producing anti-OX40, anti-PD-L1, anti-CD4 or anti-CD8 targeting antibodies were cultured in protein-free hybridoma medium (Gibco) and antibodies were purified using a Protein G column. Cell lines were MAP-tested before the start of the study and regularly negatively tested for mycoplasma infections. Before injections cells were washed with PBS, trypsinized, washed 3 times with PBS and then injected in 200 μL PBS, supplemented with 0.2% BSA.

Mice were inoculated in the flank with 0.3 × 10^6^ MC-38 (subcutaneously, 200 μL volume) or with HCmel12 (intradermally, 30 μL volume) tumor cells. Tumor outgrowth was monitored by caliper-based measurements in three dimensions. When required, mice were allocated over the different groups such that the average tumor size on the first day of therapy was equal in all of the groups. Mice were euthanized when tumor size reached >1000 mm^3^ in volume or when mice lost >20% of their total body weight (relative to initial body mass). Treatment schedule of experiments are indicated in the respective figures and legends.

#### Antibody-based interventions *in vivo*

Antibodies targeting mouse CD8 (clone 2.43), CD4 (clone GK1.5), OX40 (clone OX86) and PD-L1 (clone MIH-5) were purified from hybridoma cultures. The agonistic OX40 antibodies (150 μg per mouse on day 7 after tumor challenge) were injected subcutaneously in the flank near the tumor along with CpG (ODN1826) (25 μg per mouse). The blocking anti-PD-L1 antibodies were administered intraperitoneally (150 μg per mouse on day 10, 13 and 17 after tumor challenge). CD8^+^ and CD4^+^ T cell depleting antibodies were administered intraperitoneally twice weekly (first injection 150 μg/mouse followed by 50 μg/mouse) for 2 weeks starting one day before tumor challenge. Anti-mouse CXCR3 (clone CXCR3-173), NKG2D (clone CX5) and NK1.1 (clone PK136) antibodies were purchased from Bio X Cell (Lebanon, NH, USA) and administered intraperitoneally (150 μg per mouse on day 7, 10, 13 and 17). Depletion and blockade of the antibodies was verified by flow cytometry.

### Method details

#### Preparation for cytometry

Peripheral blood was collected from the tail vein. Spleens, tumor-draining and non-tumor draining (inguinal) lymph nodes were minced through 70 μm cell strainers. Bone marrow cells were extracted from the femurs and tibias by flushing with medium containing 8% FBS. Erythrocytes were removed from blood and tissues using a hypotonic ammonium chloride lysis buffer for 2 min. Tumors were obtained after transcardial perfusion with 30 mL of PBS/EDTA (2 mM), and after mincing incubated with 2.5 mg/mL Collagenase D and DNAse (Roche) for 20 min at 37°C. Single-cell suspensions were obtained by using 70 μm cell strainers (BD Biosciences). All samples were washed with medium containing 8% FBS before further processing.

#### Lymph node cell numbers

Tumor-draining and non-draining inguinal lymph nodes were isolated and minced through 70 μm cell strainers. Following a brief red blood cell lysis the cells were washed and diluted in medium containing 8% FBS. Cell counts were obtained using the CytoSMART Corning Cell Counter.

#### Flow cytometry

After the single-cell suspension preparation, samples were washed with staining buffer (PBS with 2% FBS). Mouse Fc-Receptors were blocked with anti-mouse CD16/32 (clone 2.4G2) and 10% naive mouse serum for 15 min before antibody staining. Following a wash step with PBS, live/dead staining was performed using Zombie NIR (Biolegend) for 10 min at room temperature. After washing in staining buffer, cells were stained using combinations of fluorescently labeled antibodies and tetramers for at least 0.5h. MHC class I tetramers (Adpgk – H-2D^b^ tetramer containing the ASMTNMELM peptide, M8 – H-2K^b^ tetramer containing the KSPWFTTL MuLV p15E peptide, Trp2 - H-2K^b^ tetramer containing the SVYDFFVWL TRP2 peptide were all made in house and either labeled with APC or PE. Intracellular Granzyme B and ID2 stainings were performed after fixation in True-Nuclear fixation buffer for 45 min at room temperature and permeabilization in True-Nuclear permeabilization buffer. Intracellular cytokine staining was performed after incubation for 5 h with Remel PHA Purified (ThermoFisher Scientific) in the presence of 2 μg/mL Brefeldin. Samples were acquired on a BD FACS LSR Fortessa X-20 4L (BD Biosciences, San Jose, CA, USA) or a 3L Cytek Aurora flow cytometer at the Flow cytometry Core Facility of Leiden University Medical Center. The data was analyzed using FlowJo v10 (Treestar) software and OMIQ data analysis software. Cells were first gated on the basis of FSC-A and SSC-A. Next, FSC-A and FSC-H were used to gate single cells and then a fixable viability stain was used to gate on the live cells. From this population we used the marker CD45 to gate leukocytes.

#### CyTOF mass cytometry and data analysis

After the single-cell suspension preparation, debris and aggregates were removed using a 100/60/40/30-percent gradient of Percoll (GE HealthCare) in RPMI 1640 (Lonza), and pelleted single cells in the 40% fraction were resuspended. Approximately 3 × 10^6^ cells were stained for each sample. Metal-conjugated antibodies were either purchased from Fluidigm Sciences or antibodies were conjugated in-house using the MaxPar X8 antibody labeling Kit (Fluidigm Sciences) according to manufactures instructions. For all non-cadmium metals or with the Maxpar MCP9 for cadmium metals, and respectively stored in Antibody Stabilization Buffer (Candor Bioscience GmbH) or HRP-Protector peroxidase stabilizer (Boca Scientific) was used. Samples were incubated with 1 μM Cell-ID intercalator-103Rh to identify dead cells, followed by FcR blockage with mouse serum (2%) and FcR blocking anti-mouse CD16/32 antibodies (clone 2.4G2, BD Biosciences). Next, the metal-conjugated antibody mix was added, and cells were incubated overnight up to 48 h with 125 nM Cell-ID Intercalator-Ir in MaxPar Fix and Perm. Prior to acquisition on a Helios mass cytometer (Fluidigm, San Francisco, CA, USA), samples were centrifuged and resuspended in MilliQ and measured directly. Data were normalized using EQ Four Element Calibration Beads with the reference EQ passport P13H2302. Mouse CyTOF data analysis was performed by pre-gating live singlet CD45^+^ cells using FlowJo software (Tree Star), followed by non-supervised clustering based using the hierarchical tSNE (HSNE) function of Cytosplore with 5 levels. Downstream analysis was performed using Cytofast, a visualization tool previously described and used by our group.^45^^,^^46^^,^^78^^,^^82^ Briefly, data was transformed using the arcsinh function with a cofactor of 5. Next, data was clustered by FlowSOM and visualized in heatmaps. To display the data quantitatively the clusters are displayed in bar graphs. Network graphs were created with the ‘igraph’ package v1.2.11 in R software v4.1.2. Correlations between cluster frequencies were calculated using the ‘cor’ function from the ‘stats’ package v.4.0.1, using pairwise Pearson correlations.

For analysis of human CyTOF data by Cytosplore, single, live CD45^+^ cells of all samples (before and after therapy) were gated and down sampled to 80.000 leukocytes per sample in FlowJo software (Tree Star). CD45^+^ cells were sample-tagged, hyperbolic ArcSinh transformed with a cofactor of 5, and subjected to dimensionality reduction analysis in Cytosplore. Major immune lineages were identified at the overview level of a 4-level hierarchical stochastic neighbor embedding (HSNE) analysis on CD45^+^ data from all samples (2.1 × 10^6^ cells) with a default perplexity and iterations (30 and 1000, respectively).^44^ Clustering of the data was performed by Gaussian mean shift (GMS) clustering, using a sigma value of 30. Manual merging of clusters based on major lineage markers resulted in 2 major clusters: CD8 positive and CD8 negative (Figure S4). CD8^+^ T cells (in total 4.4 × 10^5^ cells) were then analyzed in a data-driven manner using t-distributed stochastic neighbor embedding (tSNE). These cells were GMS clustered using a sigma value of 19. Based on the dendrogram, clusters that showed high similarity in ArcSinh5-transformed median expression of all markers were merged, resulting in 31 clusters. Quantification of frequencies of clusters in each samples was performed in Graphpad Prism v9.3.1. For analysis with OMIQ.ai, all samples were down sampled to 4185 CD8 T cells per sample. opt-SNE and FlowSOM consensus metaclustering was performed with 24 clusters but on the basis of CD56 expression cluster 16 was split into two clusters, resulting in 25 clusters in total. opt-SNE was used to perform dimension reduction using default perplexity and iterations (30 and max 1000 respectively).

#### Single-cell RNA sequencing and data analysis

To purify mouse T cells from blood, the Pan T Cell Isolation Kit II for mouse (Miltenyi Biotec) was used two consecutive times to reach >98% T cell purity after red blood cell lysis. A consecutive step to remove debris from the blood was performed by using the Debris Removal Solution (Miltenyi Biotec) according to the manufacturer protocol. Next, cells were subjected to single-cell-RNA-sequencing. Droplet-based 3′ end massively parallel single-cell RNA sequencing (scRNAseq) was performed by encapsulating sorted live T cells into droplets and libraries were prepared using Chromium Single Cell 3′ Reagent Kits v2 according to manufacturer’s protocol (10x Genomics). The generated scRNAseq libraries were sequenced using a Illumina Hiseq4000 by GenomeScan (Leiden, The Netherlands) with a sequencing depth of at least 50,000 reads per cell.

Downstream analysis was performed using the Seurat R package.^83^ Briefly, for each sample, mitochondrial, ribosomal and hemoglobin genes were excluded. Further, cells expressing less than 200 genes, and genes that were expressed in less than 3 cells were excluded. Next, all samples were pooled together into one dataset, and outlier cells expressing more than 2900 genes were excluded, which resulted in a dataset of 5260 cells. Next, the dataset was log1p normalized with a scaling factor of 10,000. Next, the set of 1709 highly variable genes were selected for further analysis. Dataset was preprocessed using principal component analysis. Using the top 15 principle components, the dataset was clustered using Louvain (graph-based community detection) and visualized using tSNE. Differentially expressed genes were identified between different cell groups, using Wilcoxon rank-sum test with Bonferroni multiple test correction (adjusted P-value <0.05). Within the CD4^+^ and CD8^+^ T cell populations, cells expressing the *Id2*, *Klrk1* and *Klrc1* genes were compared. DE genes were obtained between positive and negative groups of cells expressing these genes (expression >1 was considered positive, otherwise negative). In the volcano plots the log2 fold change (FC) in gene expression on the x axis and unadjusted p values on the y axis are illustrated. Black dots represent genes with adjusted P-value >0.05, red dots represents genes with adjusted P-value <0.05 and absolute average log2 FC < 1, green dots with gene name represent genes with adjusted P-value <0.05 and absolute average log2 FC > 1.

#### Bulk RNA sequencing and data analysis

Splenic CD43^1B11+^ and CD43^1B11−^ CD8^+^ T cells from PDOX treated mice were FACS sorted using the BD FACSAria. RNA was isolated using the Nucleospin RNA Mini Kit (Macherey-Nagel) and rRNA-depletion was performed using FastSelect HRM probes (Qiagen). Stranded libraries were prepared using Superscript III (Invitrogen) followed by adapter ligation using the Kapa Hyper Prep Kit (Roche) and UDI-UMI adapters (IDT). Sequencing was performed on the Illumina platform Novaseq6000 by Genomescan. Raw reads quality control and filtering was performed using Cutadapt v2.4. The filtered reads were aligned to the mouse reference genome GRCm38 using STAR 2.7.3a. Umitools version 0.5.5 was used to remove UMI detected duplicate sequences. HTseq-count v0.11.2 was used to quantify the reads to gene count based on the Ensembl gene annotation version 100. Differential-expression analysis was performed in Qlucore Omics Explorer (version 3.7), using the trimmed mean of log expression ratios method (TMM).

#### Immunofluorescence

4 μm FFPE tissue sections were deparaffinized, subjected to heat induced epitope retrieval in citrate (10 mM, pH 6.0), and incubated with Super-Block (ThermoFisher Scientific) to reduce unspecific antibody binding. Assessment of CD8^+^ T cell infiltration was performed by indirect immunofluorescence detection with rabbit anti-mouse CD8 antibody (clone D4W2Z, Cell Signaling) and a goat anti-rabbit Alexa 647-labelled secondary antibody (Thermo Fisher). DAPI was used for nuclear identification. Images were acquired with the Vectra V.3.0.5 system (PerkinElmer) at 20× magnification. CD8^+^ T cells in the TME were automatically phenotyped and counted with inForm V.2.4 image analysis software (PerkinElmer), after manual training and validation of procedures.

#### Gene expression and protein interaction profiling

Gene Expression Profiling Interactive Analysis^47^^,^^48^ was used to generate the overall survival (Kaplan-Meier) and Spearman correlation data of skin cutaneous melanoma (SKCM). Overall survival of single genes was performed with GEPIA^47^ (Group cutoff: Median; Cut-off high value and low value was set to 50%; hazard ratio: Yes; confidence interval: 95%). Using the GEPIA2 computation work-flow,^48^ which is based on the UCSC Xena project (http://xena.ucsc.edu), we performed survival analysis on gene signatures that were derived from the mass cytometry data; Gene signature cluster 2: CD45, CD3e, CD8a, KLRG1, KLRB1 (CD161), CD44, and ITGB1 (CD29); Gene signature cluster 7: CD45, CD3e, CD8a, KLRG1, CD44, NCAM1 (CD56), ITGB1; Gene signature cluster 8: CD45, CD3e, CD8a, KLRG1, CD44 and ITGB1. The search tool for retrieval of interacting genes (STRING) was applied to predict functional interactions of proteins.^84^

### Quantification and statistical analysis

Survival of the differentially treated animals was compared by the Kaplan-Meier method and log rank (Mantel-cox) test. Statistical analysis was performed using the Mann-Whitney or unpaired two-tailed Student’s *t* test (for 2 groups) or ANOVA(>2 group comparisons). Differences were considered statistically significant at p < 0.05.

## Data Availability

- The scRNA-seq dataset generated here is available in the SRA repository and can be accessed using GEO accession number: GSE193699 (https://www.ncbi.nlm.nih.gov/geo/query/acc.cgi?acc=GSE193699) - This paper does not report original code. - Any additional information required to reanalyse the data reported in this work paper is available from the lead contact upon request.

## Supplemental information

Document S1. Figures S1–S7

Document S2. Article plus supplemental information
