# Supplementary material for: OX40 agonism enhances PD-L1 checkpoint blockade by shifting the cytotoxic T cell differentiation spectrum
Source: Cell Rep Med. 2023 Feb 15;4(3):100939. doi: 10.1016/j.xcrm.2023.100939 (PMC10040386; doi:10.1016/j.xcrm.2023.100939)
Supplement: Document S1. Figures S1–S7 [file mmc1.pdf]

Cell Reports Medicine, Volume *4*

Supplemental information

OX40 agonism enhances PD-L1 checkpoint blockade by shifting the cytotoxic T cell differentiation spectrum

Tetje C. van der Sluis, Guillaume Beyrend, Esmé T.I. van der Gracht, Tamim Abdelaal, Simon P. Jochems, Robert A. Belderbos, Thomas H. Wesselink, Suzanne van Duikeren, Floortje J. van Haften, Anke Redeker, Laura F. Ouboter, Elham Beyranvand Nejad, Marcel Camps, Kees L.M.C. Franken, Margot M. Linssen, Peter Hohenstein, Noel F.C.C. de Miranda, Hailiang Mei, Adriaan D. Bins, John B.A.G. Haanen, Joachim G. Aerts, Ferry Ossendorp, and Ramon Arens

Supplemental Information

OX40 agonism enhances PD-L1 checkpoint blockade by shifting the cytotoxic T cell differentiation spectrum

Tetje C. van der Sluis, Guillaume Beyrend, Esmé T.I. van der Gracht, Tamim Abdelaal, Simon P. Jochems, Robert A. Belderbos, Thomas H. Wesselink, Suzanne van Duikeren, Floortje J. van Haften, Anke Redeker, Laura F. Ouboter, Elham Beyranvand Nejad, Marcel Camps, Kees LMC Franken, Margot M. Linssen, Peter Hohenstein, Noel F.C.C. de Miranda, Hailiang Mei, Adriaan D. Bins, John B.A.G. Haanen, Joachim G. Aerts, Ferry Ossendorp, Ramon Arens

Supplemental item titles

Figure S1. Comparison of immunotherapeutic strategies on tumor growth and circulating immune cell subsets and identification of transcriptional changes in therapy-responsive T cell subsets in the blood circulation. Related to Figure 1.

Figure S2. Circulating immunotherapy-responsive T cell subsets express increased NK cell receptors and migration-associated cell surface molecules. Related to Figure 2.

Figure S3. Systemic activation of immunotherapy-responsive $CD4^+$ and $CD8^+$ T cells. Related to Figure 4.

Figure S4. Identification of NK cell receptor expressing $CD8^+$ T cell subsets in the blood circulation of PD-1 therapy-responsive patients. Related to Figure 5.

Figure S5. Increased NK cell receptor expressing $CD8^+$ T cell subsets in the blood circulation of PD-1 therapy-responsive patients. Related to Figure 5.

Figure S6. Elevated therapy-responsive T cell subsets in the blood circulation associate to increased presence in tumor-microenvironment and draining-lymph nodes. Related to Figure 6.

Figure S7. CXCR3 expression empowers tumor migration of therapy-responsive T cell subsets. Related to Figure 7.

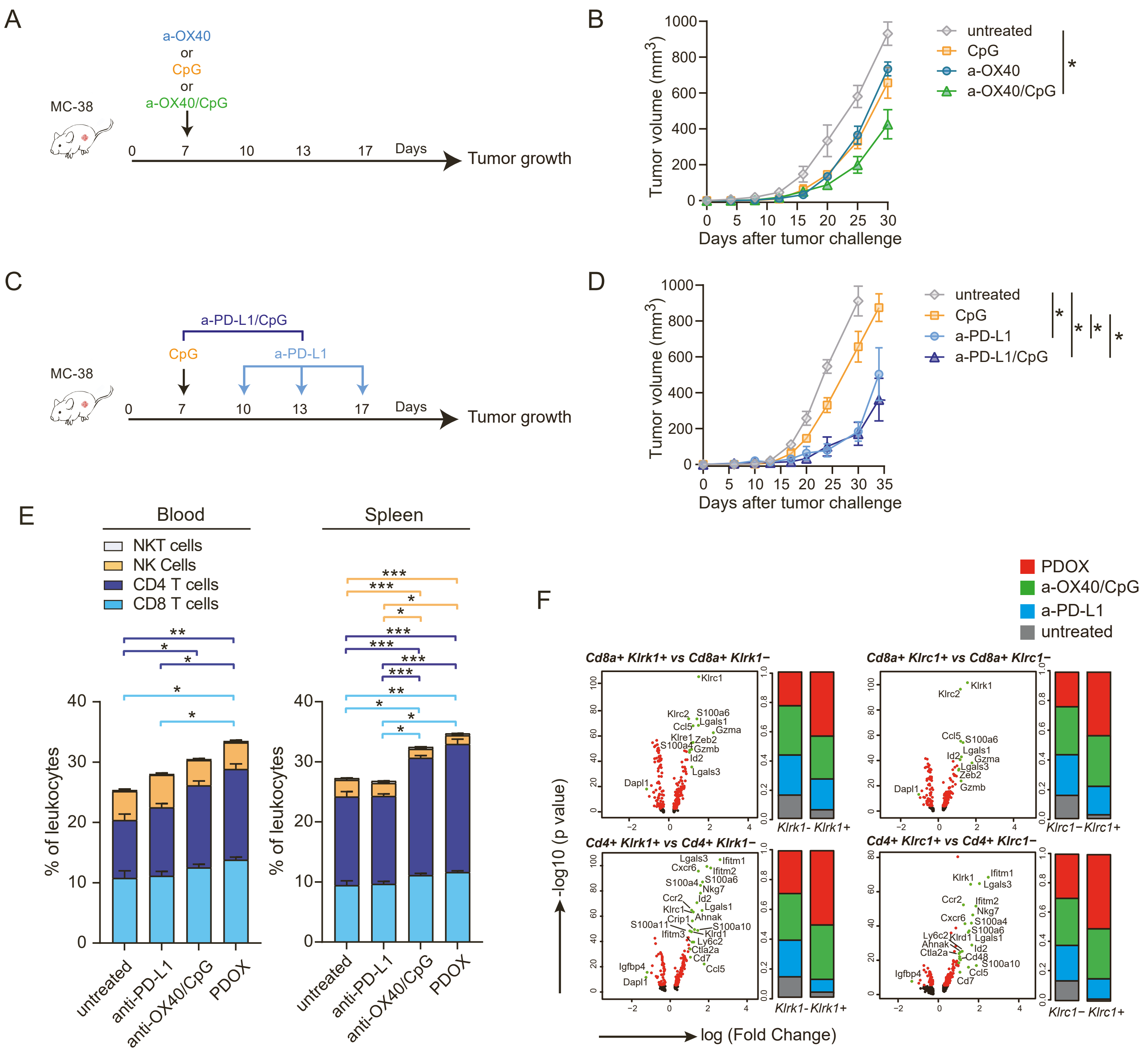

Figure S1. Comparison of immunotherapeutic strategies on tumor growth and circulating immune cell subsets and identification of transcriptional changes in therapy-responsive T cell subsets in the blood circulation. Related to Figure 1. (**A**) Schematic of the strategy. WT mice were subcutaneously challenged with MC-38 syngenic tumors and were left untreated or treated with anti-OX40, CpG or anti-OX40/CpG. (**B**) Tumor growth of untreated and treated mice. (**C**) Schematic of the strategy. WT mice were subcutaneously challenged with MC-38 syngenic tumors and were left untreated or treated with anti-PD-L1, CpG or anti-PD-L1/CpG. (**D**) Tumor growth of untreated and treated mice. (**E**) The percentage of $CD8^+$ T cells, $CD4^+$ T cells, NK cells and NKT cells within the blood circulating leukocytes (live/$CD45^+$) (left) and spleen (right). (**F**) Volcano plots showing significant gene expression related to *Klrk1* and *Klrc1* expression in $CD4^+$ and $CD8^+$ T cells. The log2 fold change (FC) in gene expression on the x-axis and unadjusted P values on the y-axis are illustrated. Black dots represent genes with adjusted P-value > 0.05, red dots represents genes with adjusted P-value < 0.05 and absolute average log2 FC < 1, green dots with gene name represent genes with adjusted P-value < 0.05 and absolute average log2 FC > 1. Bar graphs indicate the percentage of the cell origin according to their treatment.

Data shown in (E) and (F) are collected from mice at day 18 post tumor challenge. p-values in (B), (D) and (E) were calculated by ANOVA; *p<0.05, **p<0.01, ***p<0.001. Data in (B), (D) and (E) are presented as mean ± SEM. Data shown in (B) and (D) are representative of 2 independent experiments.

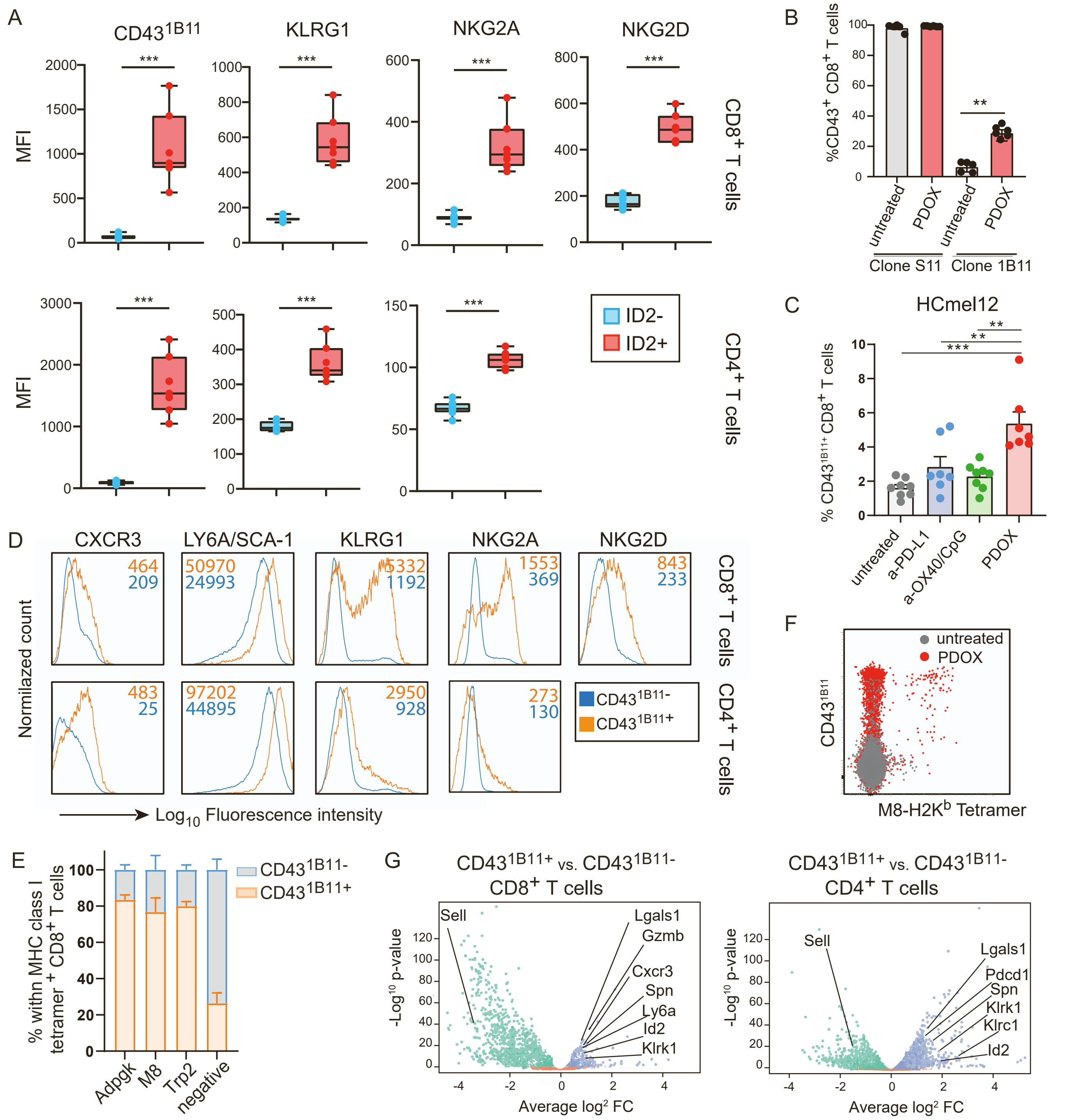

Figure S2. Circulating immunotherapy-responsive T cell subsets express increased NK cell receptors and migration-associated cell surface molecules. Related to Figure 2. (**A**) Mean Fluorescence Intensity (MFI) of CD43^{1B11}, KLRG1, NKG2A and NKG2D expression on ID2 negative and positive cells of PDOX treated animals. Values relate to Figure 2A. (**B**) Percentage of CD43^{S11+} and CD43^{1B11+} cells within CD8^{+} T cells in blood of untreated and PDOX treated mice. (**C**) Percentage of CD43^{1B11+} cells within the total CD8^{+} T cell population in blood of untreated and ICT treated groups. (**D**) Representative histograms of marker expression in blood circulating CD43^{1B11+} and CD43^{1B11-} CD8^{+} and CD43^{1B11+} and CD43^{1B11-} CD4^{+} T cell populations of MC-38 challenged PDOX treated mice. Values indicate the average MFI. (**E**) Bar graphs indicate the proportion of CD43^{1B11-} or CD43^{1B11+} cells within the Adpgk, M8, Trp2-specific CD8^{+} T cells and within the M8/Trp2-negative CD8^{+} T cells in blood after MC-38 or HCmel12 tumor challenged mice treated with PDOX. (**F**) Representative flow cytometry plot of CD43^{1B11} expression on blood circulating M8-specific CD8^{+} T cells in HCmel12 tumor bearing mice. (**G**) Volcano plots of RNA-seq data of sorted CD43^{1B11+} and CD43^{1B11-} CD8^{+} (left) and CD43^{1B11+} and CD43^{1B11-} CD4^{+} T cells (right) isolated from spleens of wild-type mice challenged with MC-38 and treated with PDOX.

Data shown in (A)-(G) are collected from mice at day 18 post tumor challenge. p-values in (A) and (B) were calculated by unpaired student's t test and in (C) by ANOVA; $^{*}p<0.05$, $^{**}p<0.01$, $^{***}p<0.001$. Data in (B), (C) and (E) are presented as mean + SEM and each dot in (A)-(C) represents an individual mouse. Data shown in (A)-(F) are representative of 2 independent experiments.

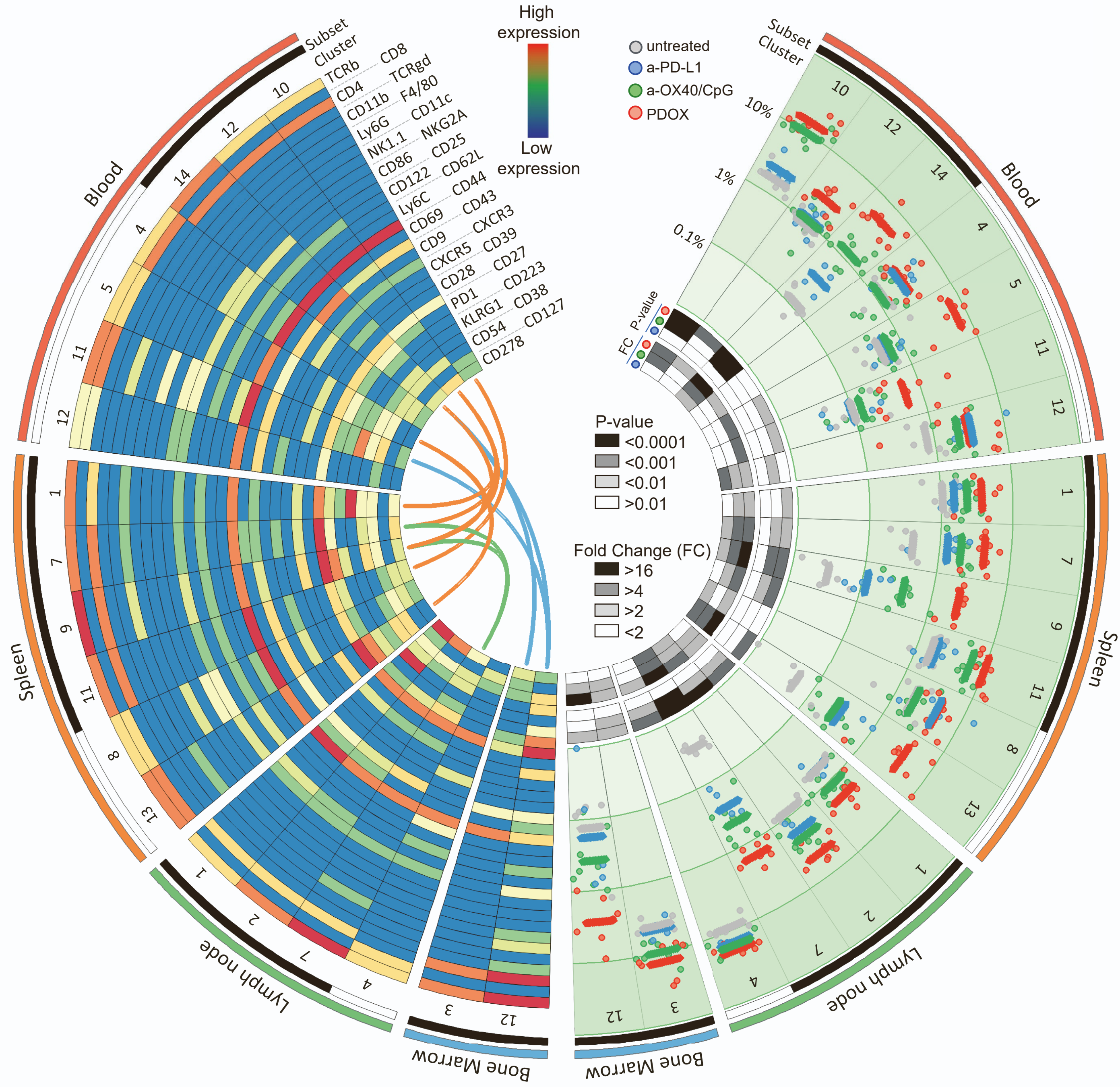

Figure S3. Systemic activation of immunotherapy-responsive CD4$^+$ and CD8$^+$ T cells. Related to Figure 4. Circos plot showing significantly different T cell clusters in blood, spleen, tumor-draining lymph nodes and bone marrow measured by CyTOF mass cytometry. Clusters are mirrored with phenotypic information on the left and abundance on the right. From outside in: tissues are depicted in coloured bars, CD8$^+$ and CD4$^+$ T cell subsets as white and black bars, respectively. Significantly different cell clusters are indicated with numbers. On the left, a heatmap for 31 relevant markers depicts normalized expression for each marker per cluster. Clusters that are highly correlated (r>0.7) between compartments are connected by coloured lines. On the right, frequency of subsets within the CD8$^+$ and CD4$^+$ T cell subsets is shown per cluster on a log-scale. Each dot represents an individual mouse. Mean values per group are indicated by lines. Multiple-testing corrected p-values for each of the ICT treated groups compared to the untreated group are shown, with p>0.01 (white), p<0.01 (light grey), p<0.001 (dark grey), p<0.0001 (black). P-values were based on t-test on log-transformed frequencies and corrected by Benjamini-Hochberg correction. Means of fold change (FC) of log-transformed values of ICT groups over the untreated group are shown with FC<2 (white), FC>2 (light grey), FC>4 (dark grey), FC>16 (black).

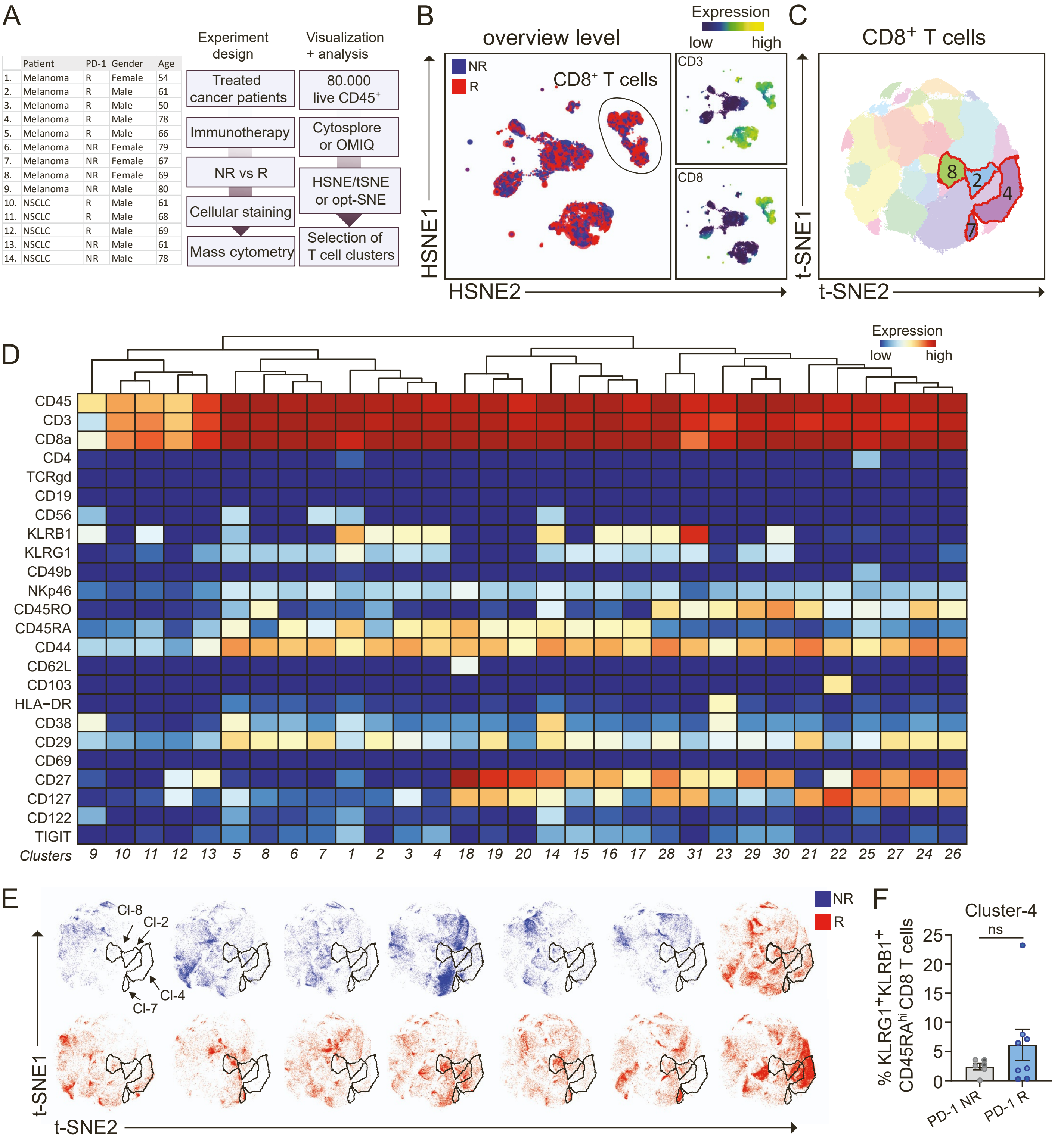

Figure S4. Identification of NK cell receptor expressing CD8^{+} T cell subsets in the blood circulation of PD-1 therapy-responsive patients. Related to Figure 5. (**A**) Schematic of the mass cytometric analysis of blood lymphocytes of cancer patients. (**B**) Left: HSNE embedding showing 1.1×10^{4} landmarks representing live CD45^{+} cells (2.1×10^{6} cells) from samples of responders (red) and non-responders (blue). CD8^{+} T cells were selected for downstream analyses. Right: marker expression of CD3 and CD8 (color indication: Blue, low expression; yellow, high expression). (**C**) Data level tSNE plot of the cluster partitions. Cluster 2, 4, 7 and 8 are highlighted. (**D**) Heat map of all 31 clusters showing median marker expression values with the corresponding dendrogram on top. Markers important for the embedding are displayed. Color indication: Blue, low expression; red, high expression. (**E**) tSNE plots of individual patients (non-responders (NR) in blue, responders (R) in red) after therapy. (**F**) Percentage of cells in cluster 4 within the total CD8^{+} T cell pool is displayed. Data are from samples collected at 2-weeks post PD-1 therapy and represented as mean ± SEM. Circles represent individual samples of non-responder melanoma (light grey), responder melanoma (light blue), non-responder lung cancer (dark grey) and responder lung cancer (dark blue) patients.

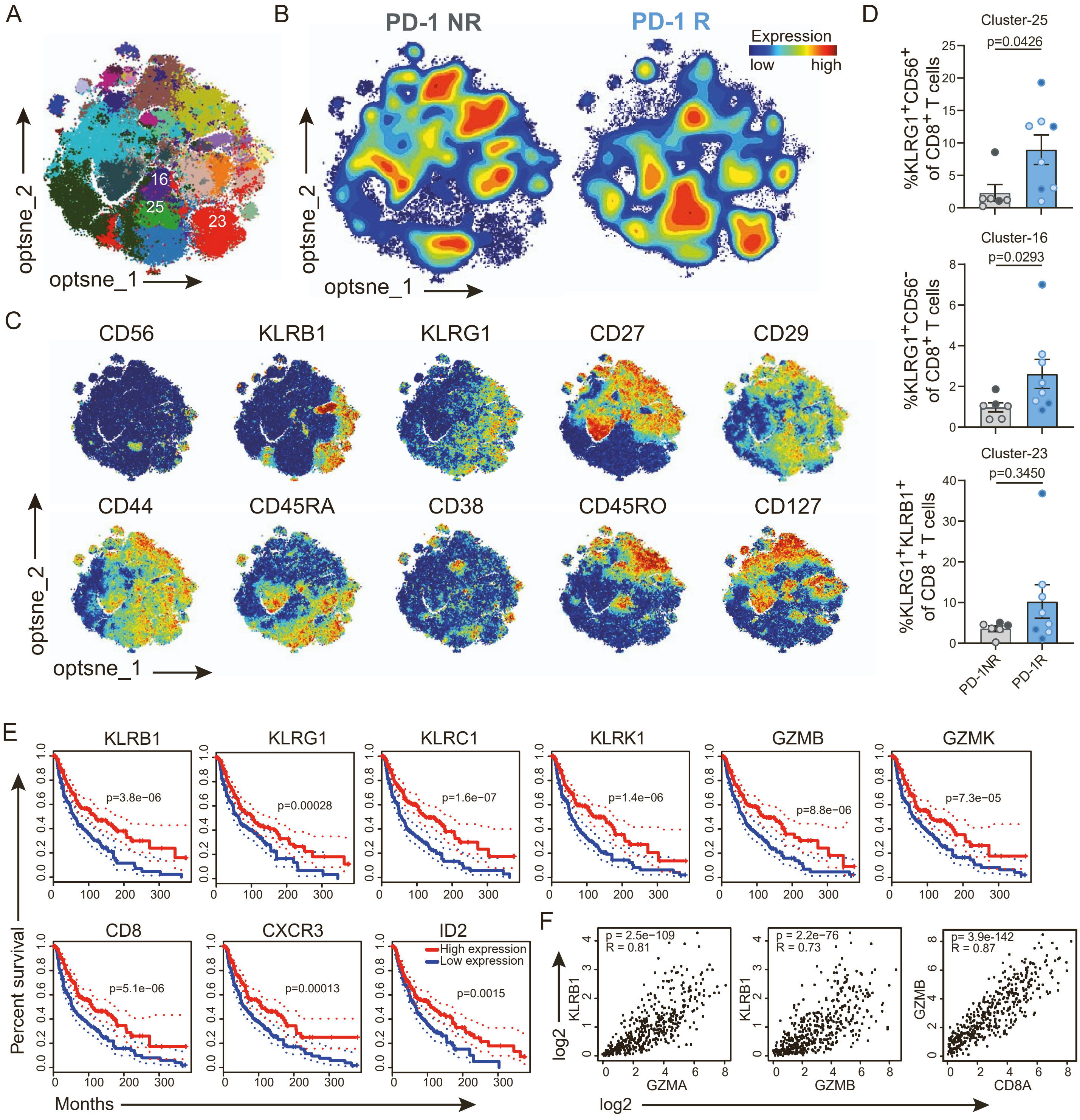

Figure S5. Increased NK cell receptor expressing CD8⁺ T cell subsets in the blood circulation of PD-1 therapy-responsive patients. Related to Figure 5. (**A-D**) All samples were down-sampled to 4185 CD8⁺ T cells per sample. opt-SNE and FlowSOM consensus metaclustering was performed with 25 clusters. Shown is an opt-SNE embedding with (**A**) the overlay of all 25 FlowSOM clusters. (**B**) Contour plot of Non-Responder (left) and Responder (right) patients. (**C**) Expression intensity of specific cell surface markers. Color indication: blue, low expression; red, high expression). (**D**) Percentage of cells in clusters 25, 16 and 23 within the total CD8⁺ T cell pool. Data are from samples collected at 2-weeks post PD-1 therapy and represented as mean ± SEM. Circles represent individual samples of non-responder melanoma (light grey), responder melanoma (light blue), non-responder lung cancer (dark grey) and responder lung cancer (dark blue) patients. p-values were calculated by Mann-Whitney U test. (**E**) Overall survival plots for high versus low marker gene expression for skin cutaneous melanoma (SKCM). Logrank p-values are indicated. (**F**) Spearman correlation analysis of therapy-responsive marker genes for SKCM. Spearman correlation coefficient and p-values are indicated.

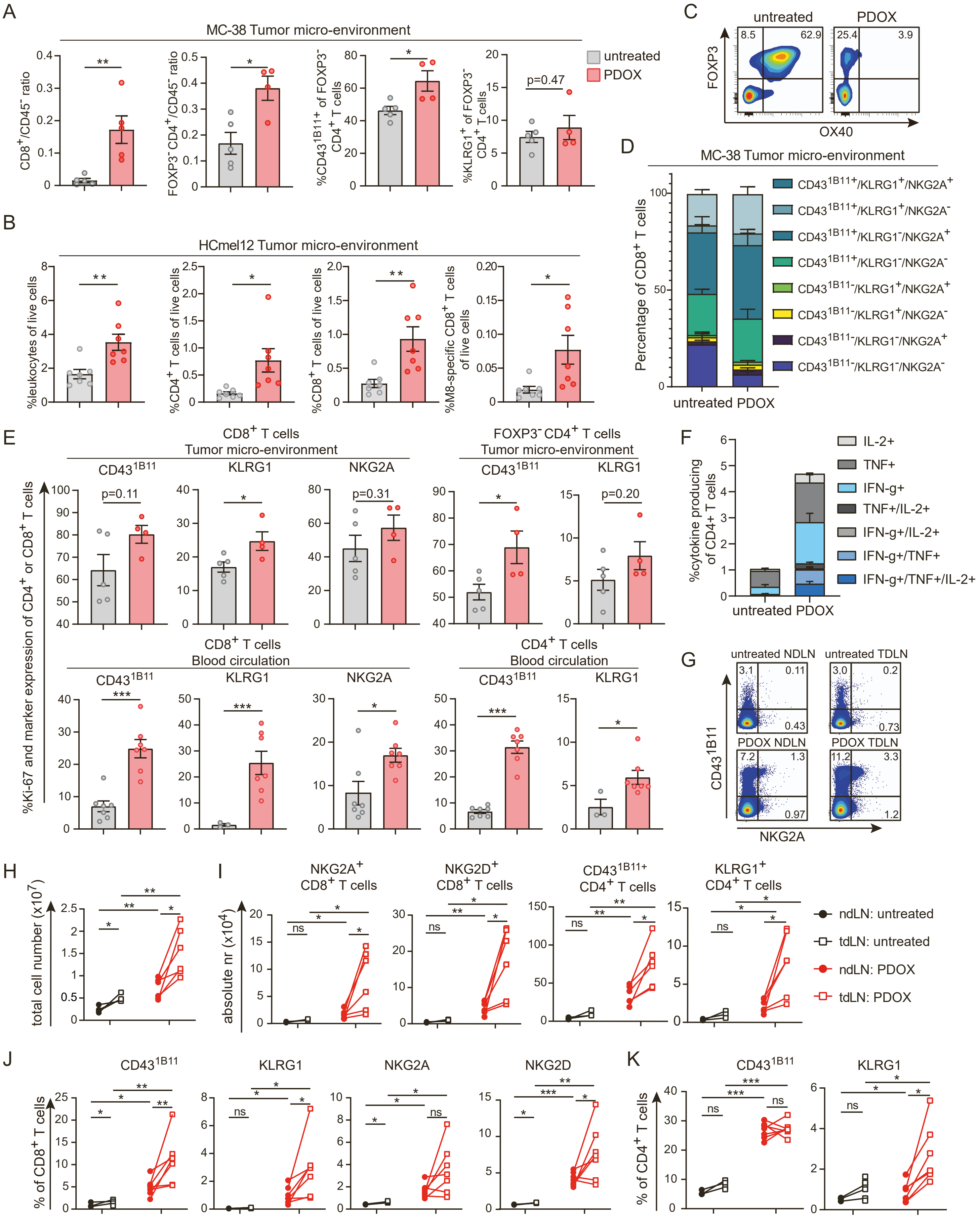

A
MC-38 Tumor micro-environment
CD8+/CD45- ratio
FOXP3- CD4+/CD45- ratio
%CD43 1B11+ of FOXP3- CD4+ T cells
%KLRG1+ of FOXP3- CD4+ T cells
p=0.47
untreated
PDOX
C
untreated
PDOX
FOXP3
OX40
8.5
62.9
25.4
3.9
B
HCmel12 Tumor micro-environment
%leukocytes of live cells
%CD4+ T cells of live cells
%CD8+ T cells of live cells
%M8-specific CD8+ T cells of live cells
D
MC-38 Tumor micro-environment
Percentage of CD8+ T cells
CD43 1B11+/KLRG1+/NKG2A+
CD43 1B11+/KLRG1+/NKG2A-
CD43 1B11+/KLRG1-/NKG2A+
CD43 1B11+/KLRG1-/NKG2A-
CD43 1B11-/KLRG1+/NKG2A+
CD43 1B11-/KLRG1+/NKG2A-
CD43 1B11-/KLRG1-/NKG2A+
CD43 1B11-/KLRG1-/NKG2A-
untreated PDOX
E
CD8+ T cells
Tumor micro-environment
FOXP3- CD4+ T cells
Tumor micro-environment
CD43 1B11
KLRG1
NKG2A
p=0.11
p=0.31
p=0.20
%Ki-67 and marker expression of CD4+ or CD8+ T cells
CD8+ T cells
Blood circulation
CD4+ T cells
Blood circulation
F
%cytokine producing of CD4+ T cells
IL-2+
TNF+
IFN-g+
TNF+/IL-2+
IFN-g+/IL-2+
IFN-g+/TNF+
IFN-g+/TNF+/IL-2+
untreated PDOX
G
untreated NDLN
untreated TDLN
PDOX NDLN
PDOX TDLN
3.1
0.11
0.43
3.0
0.2
0.73
7.2
1.3
0.97
11.2
3.3
1.2
CD43 1B11
NKG2A
H
total cell number (x10^7)
I
NKG2A+ CD8+ T cells
NKG2D+ CD8+ T cells
CD43 1B11+ CD4+ T cells
KLRG1+ CD4+ T cells
absolute nr (x10^4)
ndLN: untreated
tdLN: untreated
ndLN: PDOX
tdLN: PDOX
J
CD43 1B11
KLRG1
NKG2A
NKG2D
% of CD8+ T cells
K
CD43 1B11
KLRG1
% of CD4+ T cells

Figure S6. Elevated therapy-responsive T cell subsets in the blood circulation associate to increased presence in tumor-microenvironment and draining-lymph nodes. Related to Figure 6. (**A**) Left plots: Ratio of $CD8^+$ or $FOXP3^-$ $CD4^+$ T cells divided $CD45^-$ tumor cells. Right plots: Percentage of $CD43^{1B11+}FOXP3^-$ and $KLRG1^+FOXP3^-$ $CD4^+$ T cells out of total $CD4^+$ T cells in the TME. (**B)** Percentage leukocytes, total $CD4^+$ and $CD8^+$ T cells of live cells and percentage M8-specific $CD8^+$ T cells in the HCmel12 tumor micro-environment (TME) of untreated and PDOX treated mice. (**C**) Representative flow cytometry plots indicate FOXP3 versus OX40 expression of MC-38 tumor-infiltrated $CD4^+$ T cells. Numbers indicate the average percentage in each quadrant. (**D**) Stacked bar graph representing $CD43^{1B11}$, NKG2A and KLRG1 (co-)expression on tumor-infiltrating $CD8^+$ T cells of untreated (n=5) and PDOX treated mice (n=4). (**E**) Percentage of Ki-67 and marker (KLRG1, $CD43^{1B11}$ or NKG2A) expression (double positive cells) of tumor-infiltrating $CD8^+$ or $FoxP3^-CD4^+$ T cells, and of $CD8^+$ and $CD4^+$ T cells in the blood circulation. (**F**) The proportion of single, double and triple cytokine producing cells within the tumor-infiltrating $CD4^+$ T cells of untreated and PDOX treated MC-38 tumor challenged mice. (**G**) Representative plots of $CD8^+$ T cells in tdLNs and ndLNs of untreated and PDOX treated mice showing $CD43^{1B11}$ versus NKG2A expression. (**H**) Total cell numbers of non-draining lymph nodes (ndLN, closed circles and tumor draining lymph nodes (tdLN, open squares) of untreated or PDOX treated MC-38 tumor bearing mice. (**I**) Total cell numbers of NKG2A and NKG2D positive $CD8^+$ T cells, and $CD43^{1B11}$ and KLRG1 positive $CD4^+$ T cells in ndLN and tdLN of untreated or PDOX treated MC-38 tumor bearing mice. (**J**) Percentage of $CD43^{1B11}$, $KLRG1^+$, $NKG2A^+$ and $NKG2D^+$ $CD8^+$ T cells in ndLN and tdLN of untreated or PDOX treated MC-38 tumor bearing mice. (**K**) Percentage of $CD43^{1B11+}$ and $KLRG1^+$ $CD4^+$ T cells in ndLN and tdLN of untreated or PDOX treated MC-38 tumor bearing mice. Lines in (H)-(K) connect ndLN and tdLN from the same mouse.

Data shown in (A)-(K) are collected from mice at day 20 post tumor challenge (PDOX treatment started at day 10). p-values in (A), (D), (E) and (H)-(K) were calculated by unpaired student's t test; *p<0.05, **p<0.01, ***p<0.001, exact p-value is indicated in (A) and (E) when a p-value was >0.05, ns represents non-significant in (I), (J), and (K). Data in (A), (C), and (D)-(F) are presented as mean ± SEM and each dot in (A), (D), and (E) represents an individual mouse. Data shown are representative of 2 independent experiments.

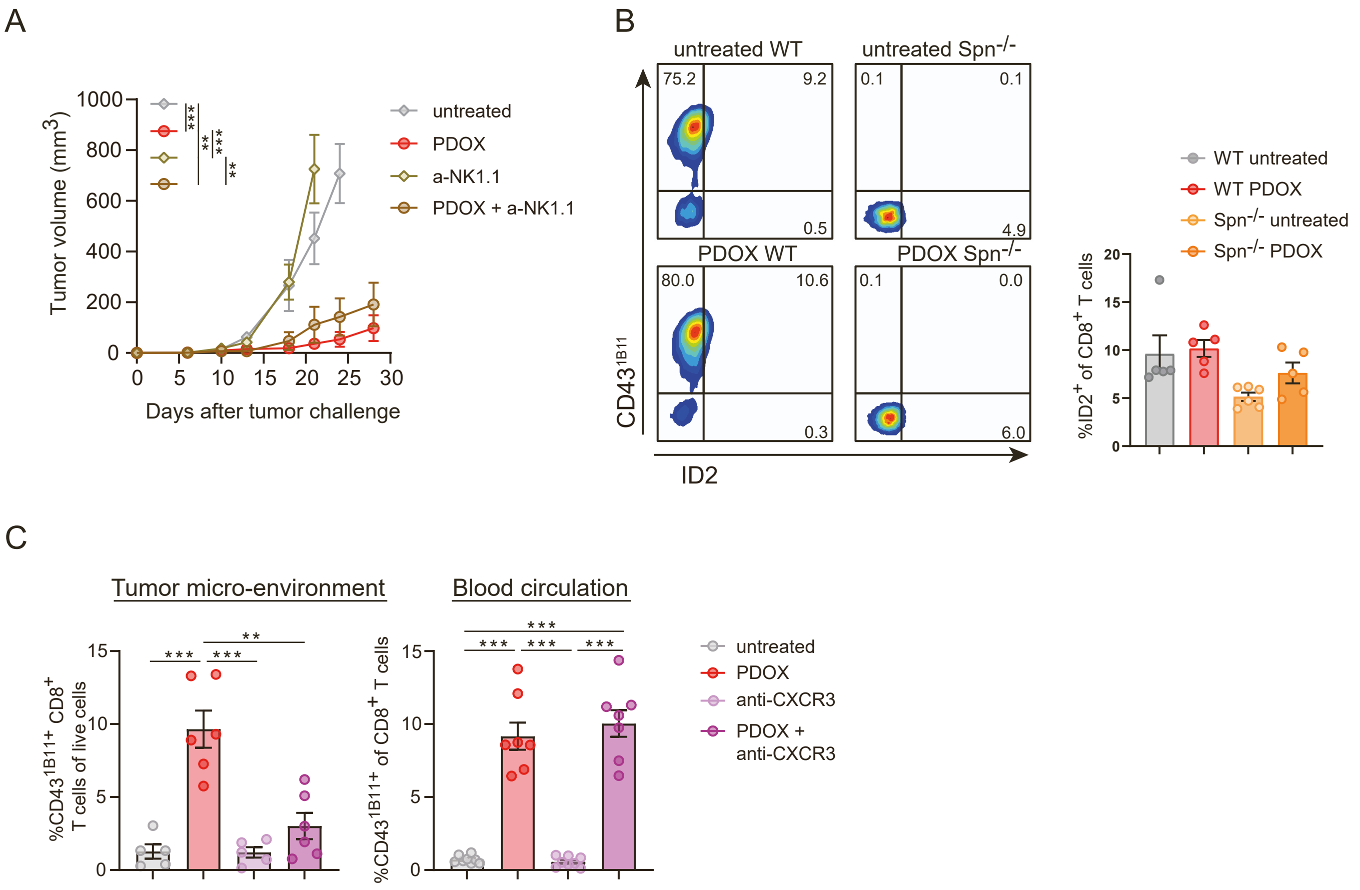

Figure S7. CXCR3 expression empowers tumor migration of therapy-responsive T cell subsets. Related to Figure 7. (**A**) Tumor growth of untreated and PDOX treated MC-38 tumor bearing mice in combination with anti-NK1.1 antibodies. (**B**) Left: Representative plots showing CD43^{1B11} versus ID2 expression of MC-38 tumor-infiltrated CD8^{+} T cells of untreated and PDOX treated wild-type and Spn$^{-/-}$ mice. Right: Percentage of ID2^{+} cells within the tumor-infiltrated CD8^{+} T cell population. (**C**) Percentage CD43^{1B11+}CD8^{+} T cells out of live cells in the MC-38 tumor-micro-environment and percentage CD43^{1B11+} cells of CD8^{+} T cells in the blood of untreated and PDOX treated mice in combination with blocking CXCR3 antibodies.

Data shown in (B)-(C) are collected from mice at day 18 post MC-38 tumor challenge. p-values in (A) and (C) were calculated by ANOVA; *p<0.05, **p<0.01, ***p<0.001. Data in (A), (B), and (C) are presented as mean ± SEM and each dot in (B) and (C) represents an individual mouse.
